# An integrated bioinformatic investigation of focal adhesion-related genes in glioma followed by preliminary validation of COL1A2 in tumorigenesis

**DOI:** 10.18632/aging.204834

**Published:** 2023-06-22

**Authors:** Guojun Yao, Ling Deng, Xinquan Long, Yufan Zhou, Xiang Zhou

**Affiliations:** 1 Department of Neurosurgery, The First People’s Hospital of Fuzhou City, Fuzhou 344099, Jiangxi, P.R. China; 2 College of Nursing and Rehabilitation, Fuzhou Medical College of Nanchang University, Fuzhou 344099, Jiangxi, P.R. China

**Keywords:** focal adhesion-related genes, tumor microenvironment, immune checkpoint, prognosis, immunotherapy

## Abstract

Focal adhesions (FAs) allow cells to contact the extracellular matrix, helping to maintain tension and enabling signal transmission in cell migration, differentiation, and apoptosis. In addition, FAs are associated with changes in the tumor microenvironment (TME) that lead to malignant progression and drug resistance in tumors. However, there are still few studies on the comprehensive analysis of focal adhesion-related genes (FARGs) in glioma. Expression data and clinical information of glioma samples were downloaded from public databases. Two distinct molecular subtypes were identified based on FARGs using an unsupervised consensus clustering algorithm. A scoring system consisting of nine FARGs was constructed using integrated LASSO regression and multivariate Cox regression. It not only has outstanding prognostic value but also can guide immunotherapy of glioma patients, which was verified in TCGA, CGGA, GSE16011, and IMvigor210 cohorts. The results of bioinformatics analysis, immunohistochemistry staining, and western blotting all revealed that the expression of COL1A2 was up-regulated in glioblastoma and related to poor prognosis outcomes in patients from public datasets. COL1A2 promotes the proliferation, migration, and invasion of glioblastoma cells. A positive correlation between COL1A2 and CD8 was determined in GBM specimens from eight patients. Moreover, the results of cell co-cultured assay showed that COL1A2 participated in the killing of GBM cells by Jurkat cells. Our study indicates that the FARGs have prominent application value in the identification of molecular subtypes and prediction of survival outcomes in glioma patients. Bioinformatics analysis and experimental verification provide a direction for further research on FARGs.

## INTRODUCTION

Glioma has the highest incidence rate (70%) among intracranial primary tumors and is characterized by high malignancy and rate of recurrence [1]. In addition to conventional treatment, immunotherapy is also an option for glioma patients [2–4]. However, the treatment effect of these regimens is not optimistic, recurrence is almost inevitable [5], and the median overall survival (OS) is hardly more than 1.5 years [6, 7]. Besides intra-tumoral heterogeneity, genetic and epigenetic factors also influence patients’ response to treatment. Identifying and elucidating the intrinsic molecular mechanism of glioma provides a theoretical basis for developing more effective therapeutic schemes.

FAs allow cells to contact the extracellular matrix (ECM), helping to maintain cell tension and enabling signal transmission [8, 9] during cell migration, differentiation, and apoptosis. FA proteins are classified according to their functions [10] as integrins, FA kinases, paxillin, etc. Some of these have been testified linked to cancer progression [11–13]. However, the predictive value of FA proteins for prognosis and treatment response in cancer is still unknown.

TME is closely related to tumorigenesis and malignant progression [14]. The change in TME, which reduces the adhesion of tumor cells, has an important impact on the drug resistance of tumors [15] and can cause metastasis [16, 17] by promoting epithelial-mesenchymal transition [18–21]. As an important component of TME, macrophages account for 30% among the immune cells, which is the highest proportion of cells in the glioma [22]. Immune checkpoint blockades (ICBs) can prevent immune escape by modulating the function of T cells [23, 24]. At present, CTLA-4, PD-1, and PD-L1 are the main immunotherapeutic targets for patients with advanced tumors, although they do not always prolong the survival time of patients [23, 25]. The failure of tumor immunotherapy is thought to be related to an immunosuppressive TME and low tumor mutational burden (TMB) [26, 27]. Up to now, glioma patients have neither effective immunotherapeutic targets nor biomarkers that can effectively predict immunotherapeutic response.

In this paper, we not only identified two distinct molecular subtypes but also constructed a scoring system with outstanding clinical application value. In addition, we screened the gene COL1A2, which is up-regulated in GBM tissue and closely related to the poor prognosis of glioma patients. In addition, we conducted intensive study on the COL1A2 gene and found that it not only participates in the regulation of biological behavior of GBM but also may be a key molecule in the TME of glioma.

## RESULTS

### Molecular classification based on FARGs in TCGA cohort

**Figure 1:**
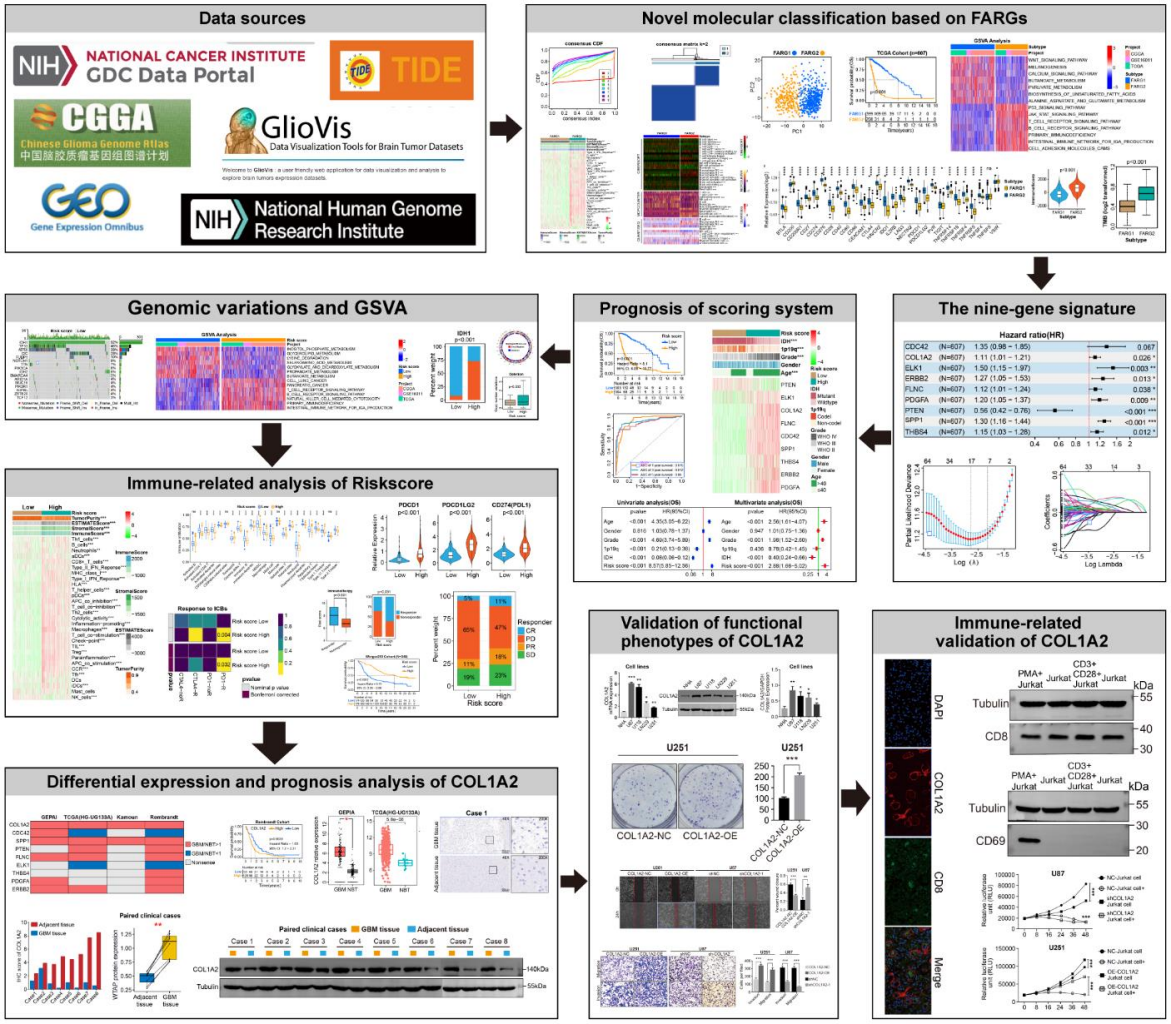
Flow diagram of this study.

**Figure 2:**
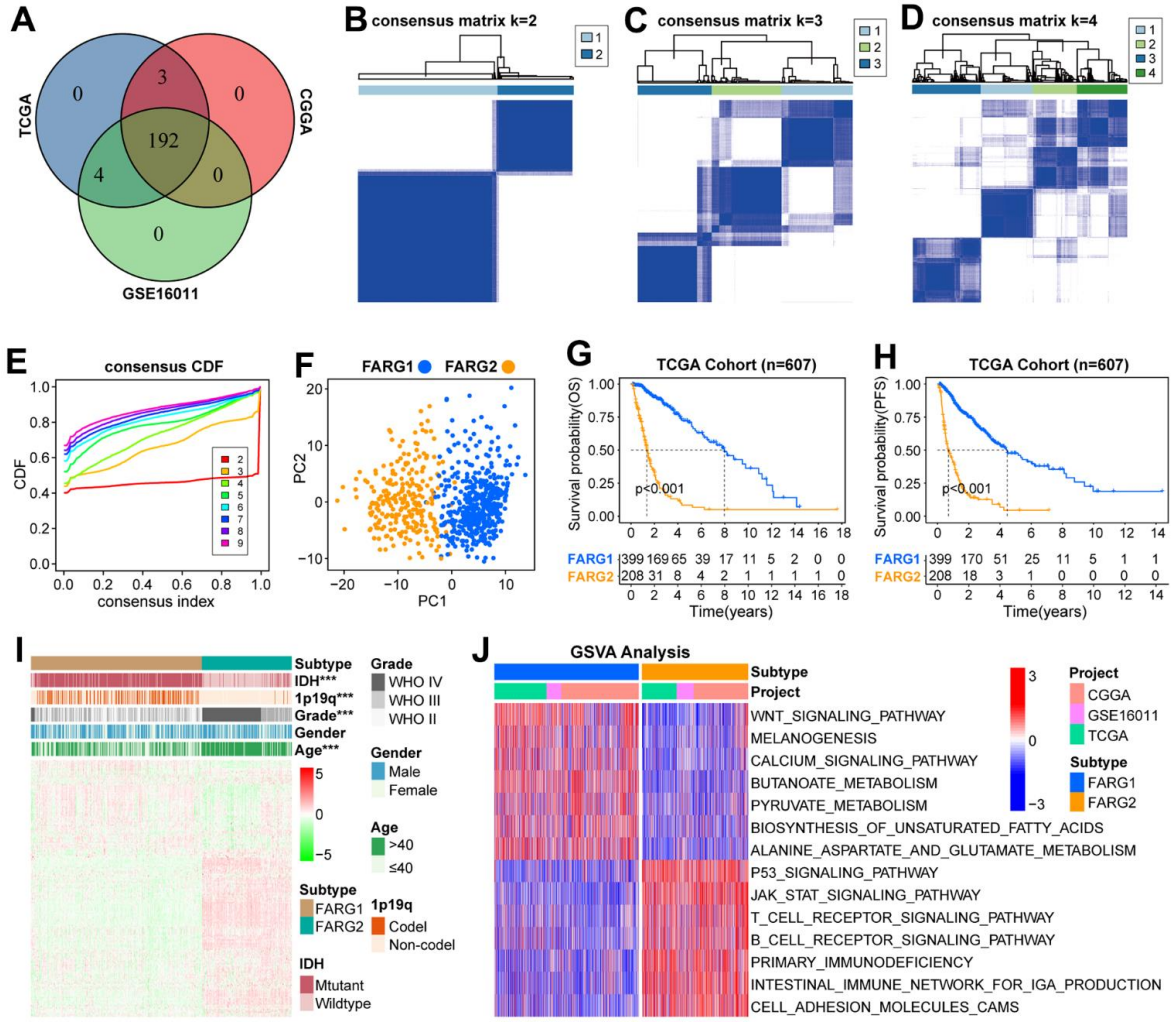
**Novel molecular classification based on FARGs in TCGA cohort.** (**A**) The intersection of 199 FARGs between TCGA, CGGA, and GSE16011 cohorts. (**B**–**D**) Consensus clustering matrix based on 192 FARGs for k=2, k=3, and k=4. (**E**) The optimal number of consensus clustering matrices is determined by the cumulative distribution function of the unsupervised consensus clustering algorithm. (**F**) Obvious differences in the transcriptomes between FARG1 and FARG2 subtypes were analyzed by principal component analysis (PCA). (**G**, **H**) K-M survival curves of glioma patients. (**I**) The distributions of five clinicopathological characteristics and 199 FARGs between FARG1 and FARG2 subtypes. (**J**) GSVA between FARG1 and FARG2 subtypes. Red and blue represent the relative activation and inhibition of the pathways, respectively. *P<0.05, **P<0.01, ***P<0.001.

First, we show the workflow of this study in Figure 1. According to the flow chart, 192 FARGs were obtained from the intersection of TCGA, CGGA, and GSE16011 cohorts (Figure 2A). A novel molecular classification was identified by using an unsupervised clustering algorithm based on the expression profiles of the 192 FARGs extracted from the TCGA cohort. According to the area under the CDF curves, probably approximately correct algorithm and correlations between clusters, we determined the optimal number of clusters is two (k=2) (Figure 2B–2E). Principal components analysis (PCA) can effectively distinguish glioma patients with the FARG1 subtype and FARG2 subtype (Figure 2F).

### Basic analysis between different subtypes in the TCGA cohort

The OS and progression-free survival (PFS) of glioma patients, low-grade glioma (LGG) patients, and GBM patients with the FARG1 subtype were significantly longer than those with the FARG2 subtype (Figure 2G, 2H). Except for gender, there are dramatic differences in the distribution of the other four clinicopathological characteristics between FARG1 and FARG2 subtypes. In addition, most FARGs are differentially expressed between the two subtypes (Figure 2I). We simultaneously performed GSVA in TCGA, CGGA, and GSE16011 cohorts and identified 167 functional pathways with different relative activity between the two subtypes (Figure 2J and Supplementary Table 3), of which 65 functional pathways were more active in FARG1 subtype, such inositol phosphate metabolism, glycerol lipid metabolism and lysine degradation, and the other 102 functional pathways were more active in FARG2 subtypes, such as cell lung cancer, pancreatic cancer, and primary immunodeficiency.

### Immune-related analysis between different subtypes in TCGA cohort

**Figure 3:**
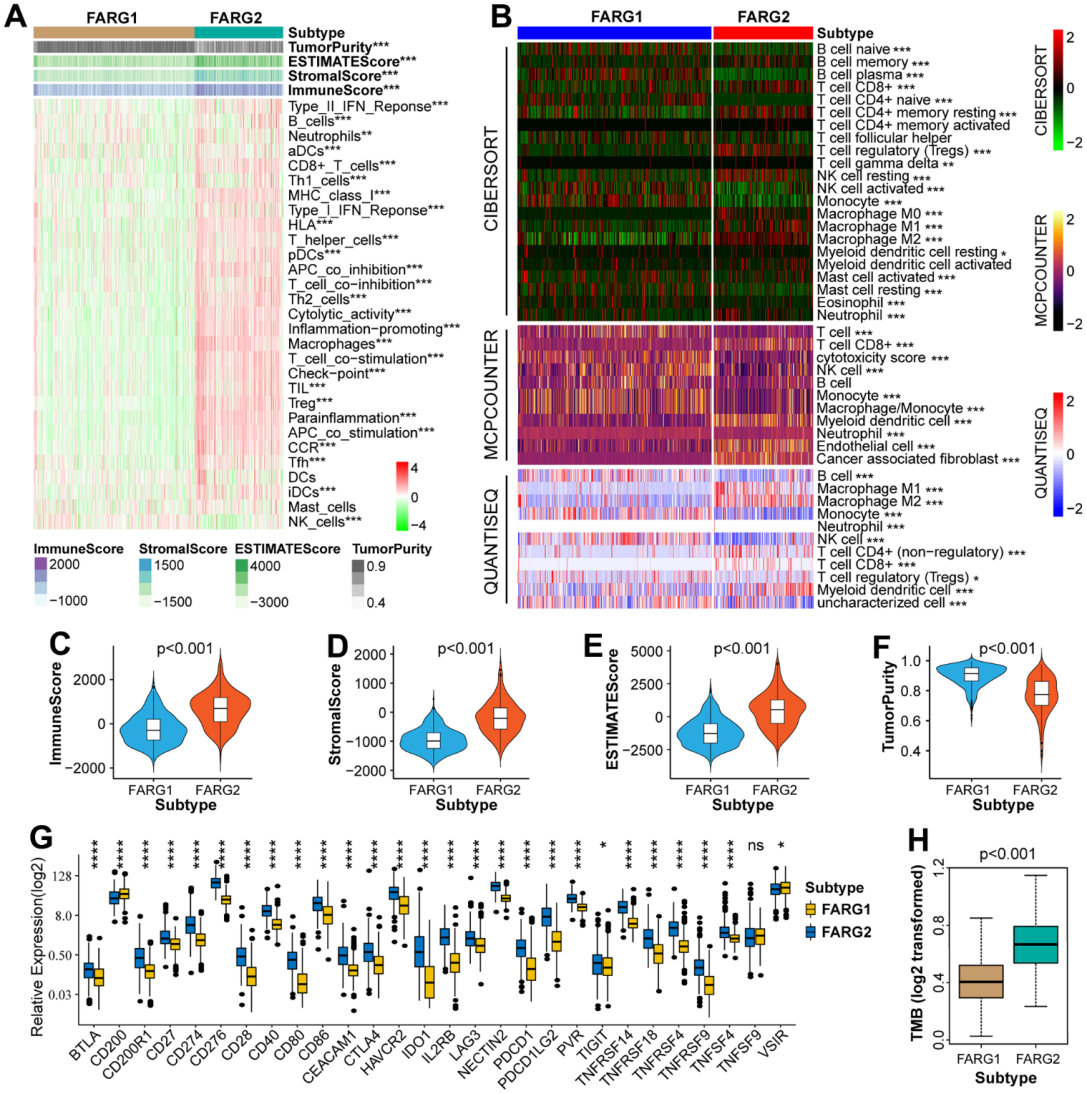
**Immune-related analysis between FARG1 and FARG2 subtypes in the TCGA cohort.** (**A**) Difference analysis of 29 immune signatures and four types of TME-related scores between FARG1 and FARG2 subtypes. (**B**) The difference in the content of immune cells was calculated by CIBERSORT, MCPCOUNTER, and QUANTISEQ algorithms between the two subtypes. (**C**–**F**) Difference analysis of TME-related scores between the two subtypes. (**G**) Difference analysis of ICPs between two subtypes. (**H**) Difference analysis of TMB between the two subtypes. *P<0.05, **P<0.01, ***P<0.001, ****p < 0.0001.

Given our above finding, we further investigated whether different subtypes have different immunological characteristics. The results revealed that there were remarkable differences in most immune signatures (except for dendritic cells and mast cells) and all four TME-related scores among the two subtypes (Figure 3A, 3C–3F). Compared with the FARG1 subtype, the glioma tissue of the FARG2 subtype contains more immune cells and stromal cells. We also estimated the content of immune cells and found that the content of almost all immune cells was different between the two subtypes (Figure 3B and Supplementary Table 4). Moreover, we also found that except for TNFSF9, other immune checkpoints (ICPs) were differentially expressed between the two subtypes of FARG1 and FARG2 (Figure 3G). Considering the effect of gene mutations on tumorigenesis and progression, we found that the TMB was dramatically different between the two subtypes (Figure 3H).

### Construction of a nine-gene scoring system

**Figure 4:**
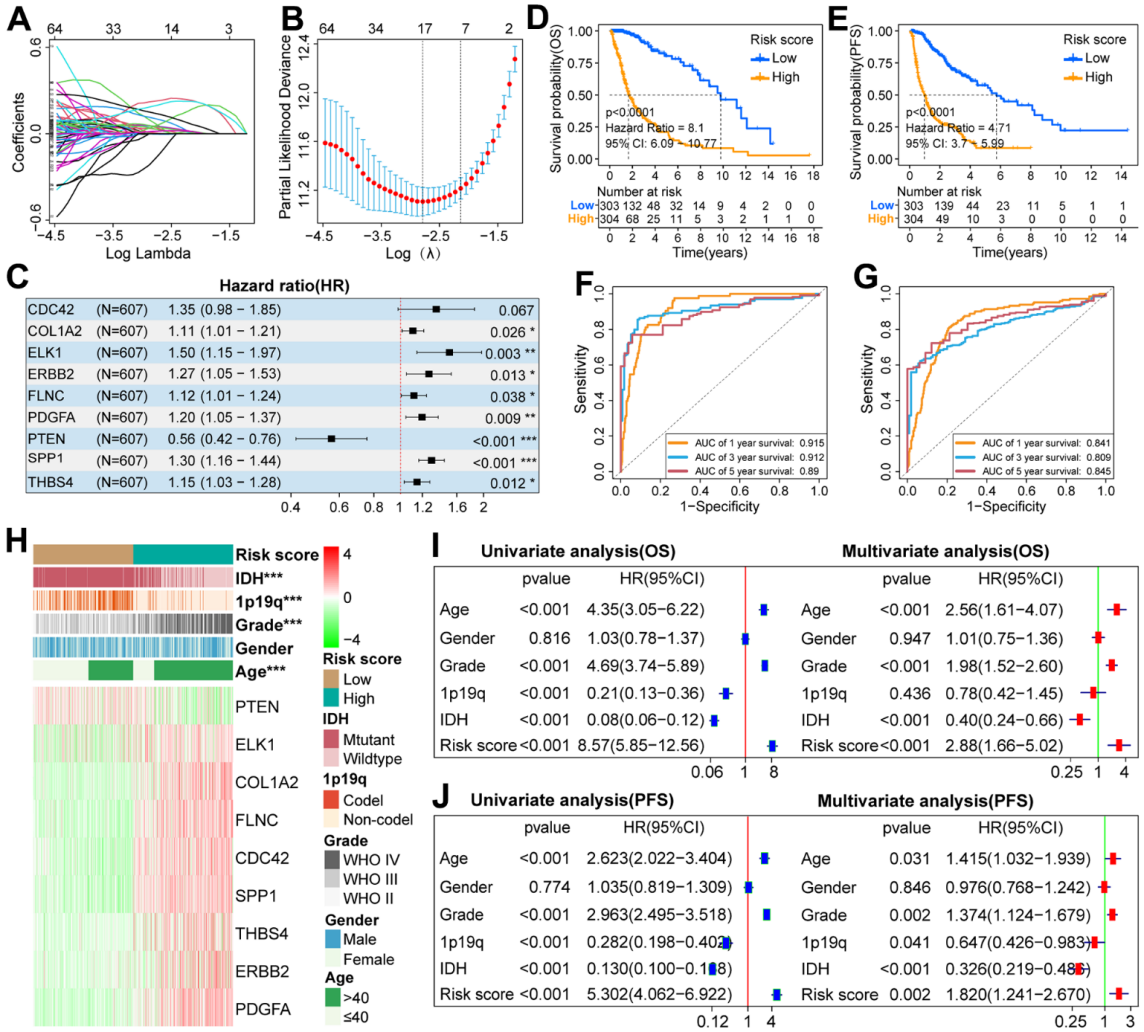
**Construction of scoring system and analysis of its prognostic value in the TCGA cohort.** (**A**) LASSO coefficient profiles of 192 FARGs. (**B**) Selection of the penalty parameter (λ) in the LASSO model via 1,000 cross-validations. The vertical dotted line passing through the red dot is drawn at the optimal value. (**C**) Nine FARGs screened by multi-Cox regression analysis were used to construct the scoring system. (**D**, **E**) The K-M survival curves showed that the OS and PFS of glioma patients in the low-risk group were longer than those in the high-risk group. (**F**, **G**) ROC curves show that risk score can effectively predict the 1,3,5-year OS and PFS for glioma patients. (**H**) The distributions of five clinicopathological characteristics and nine FARGs between high and low-risk groups. (**I**, **J**) Uni- and multi-Cox regression analysis of risk score and five clinicopathological characteristics. *P<0.05, **P<0.01, ***P<0.001.

To establish a scoring system, 175 FARGs were screened from 192 FARGs using uni-Cox regression analysis (Supplementary Table 5). These genes were further screened using the LASSO algorithm, and then 17 FARGs were obtained (Figure 4A, 4B). After that, multi-Cox regression analysis in both directions was performed and yielded nine FARGs (CDC42, COL1A2, ELK1, ERBB2, FLNC, PDGFA, PTEN, SPP1, and THBS4) (Figure 4C). The nine genes were used to construct a scoring system according to the following equation: risk score = 0.298 × (CDC42 expression) + 0.103 × (COL1A2 expression) + 0.408 × (ELK1 expression) + 0.237 × (ERBB2 expression) + 0.111 × (FLNC expression) + 0.179 × (FDGFA expression) - 0.577 × (PTEN expression) + 0.259 × (SPP1 expression) + 0.138 × (THBS4 expression).

### Prognostic analysis of scoring system

Since there are significant differences in the prognosis outcomes between GBM and LGG patients, IDH mutant and wild-type glioma patients, we should not only explore the prognostic value of the scoring system in pan-gliomas but also explore its prognostic value among different grades and different IDH mutation types. If the risk score of glioma patients was higher than the median value, they were classified as a high-risk group, otherwise, they were classified as a low-risk group. K–M survival curves showed that in the three cohorts, the OS of patients with pan-gliomas (Figure 4D and Supplementary Figure 1A, 1B), LGG (Supplementary Figure 2A, 2E, 2I), GBM (Supplementary Figure 2B, 2F, 2J), IDH mutation (Supplementary Figures 3A, 4A, 4G) and IDH wildtype (Supplementary Figures 3B, 4B, 4H) in the low-risk group tended to be longer than that of patients in the high-risk group. Moreover, the risk score can effectively predict the 1, 3, and 5-year OS rate of patients with pan-gliomas (Figure 4F and Supplementary Figure 1C, 1D), LGG (Supplementary Figure 2C, 2G, 2K), GBM (Supplementary Figure 2D, 2H, 2L), IDH mutation (Supplementary Figures 3C, 4C, 4I) and IDH wildtype (Supplementary Figures 3D, 4D, 4J) in the three cohorts (Supplementary Table 6). In addition, in the TCGA cohort, patients with pan-gliomas, IDH mutant and IDH wildtype in the low-risk group also tended to have longer PFS than patients in the high-risk group (Figure 4E and Supplementary Figure 3G, 3H), and the risk score can also effectively predict the 1, 3, 5-year PFS rate of patients with pan-gliomas, IDH mutant and IDH wildtype (Figure 4G, and Supplementary Figure 3I, 3J and Supplementary Table 6). Next, we explored the differential distribution of five clinicopathological characteristics and the differential expression of nine FARGs between the two risk groups in the three cohorts. The results showed that except for gender, there were significant differences in the distribution of the other four clinicopathological characteristics and the expression of nine FARGs between different risk groups (Figure 4H and Supplementary Figure 1E, 1G). Finally, we explored whether the scoring system can be used as an independent prognostic factor for patients with pan-gliomas, LGG, GBM, IDH mutant, or wildtype. The results showed that the independent prognostic value of risk score was superior to that of age, gender, grade, IDH status, and 1p19q status in TCGA, CGGA, and GSE16011 cohorts (Figure 4I, 4J, and Supplementary Figures 1F, 1H, 3E, 3F, 3K, 3L, 4E, 4F, 4K, 4L).

### Genomic variation analysis between high and low-risk groups

**Figure 5:**
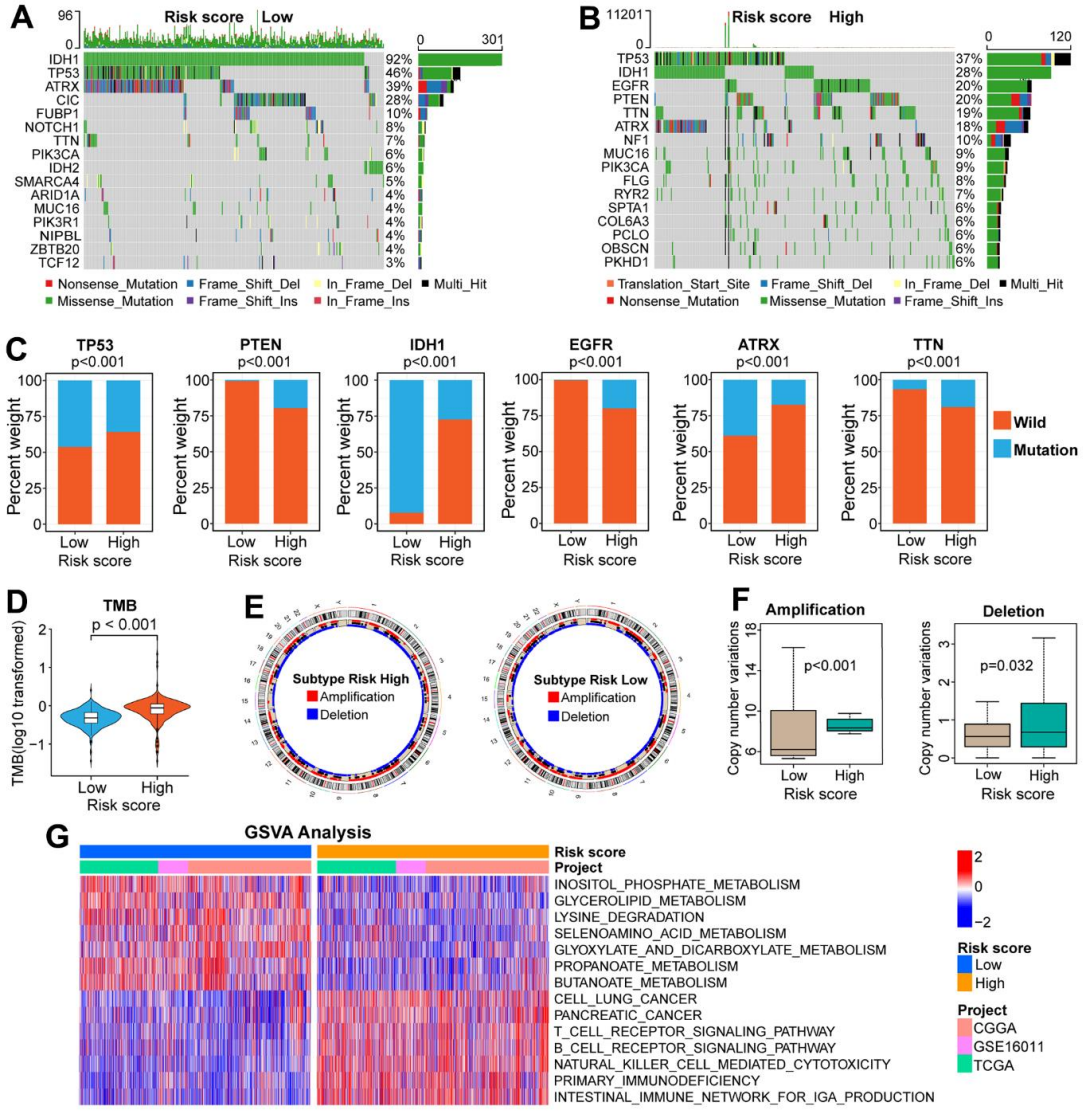
**Comparisons of genomic variations and functional annotations between high and low-risk groups in the TCGA cohort.** (**A**, **B**) Waterfall plots of the top-16 mutated genes. The genetic alteration types are listed at the bottom of the waterfall plot. The mutation frequencies of genes are listed on the right side of the waterfall Plot. (**C**) Differential distribution of mutation and wildtype of six well-known genes between the two risk groups. (**D**) Comparison of TMB between high and low-risk groups. (**E**) Circular diagram of chromosome amplification and deletion between high and low-risk groups. (**F**) CNV frequencies (amplification or deletion) were lower in the low-risk group than that in the high-risk score group. (**G**) GSVA between FARG1 and FARG2 subtypes. Red and blue represent the relative activation and inhibition of the pathways, respectively.

Because genomic variation can impact tumor immunity and immune cell infiltration patterns [28], we explored the association between genomic variation and risk score in this study. The top-16 genes with the highest mutation frequency have significant differences between the two risk groups (Figure 5A, 5B). There are also obvious differences in the distribution of mutation and wildtype of six well-known genes (TP53, PTEN, IDH1, EGFR, ATRX, TTN) between high and low-risk groups (Figure 5C). Besides, we found that glioma patients with low-risk scores inclined to have a lower TMB (Figure 5D). Moreover, patients in the low-risk group had a lower frequency of CNV, either amplification or deletion (Figure 5E, 5F).

### Immune-related analysis between high and low-risk groups

**Figure 6:**
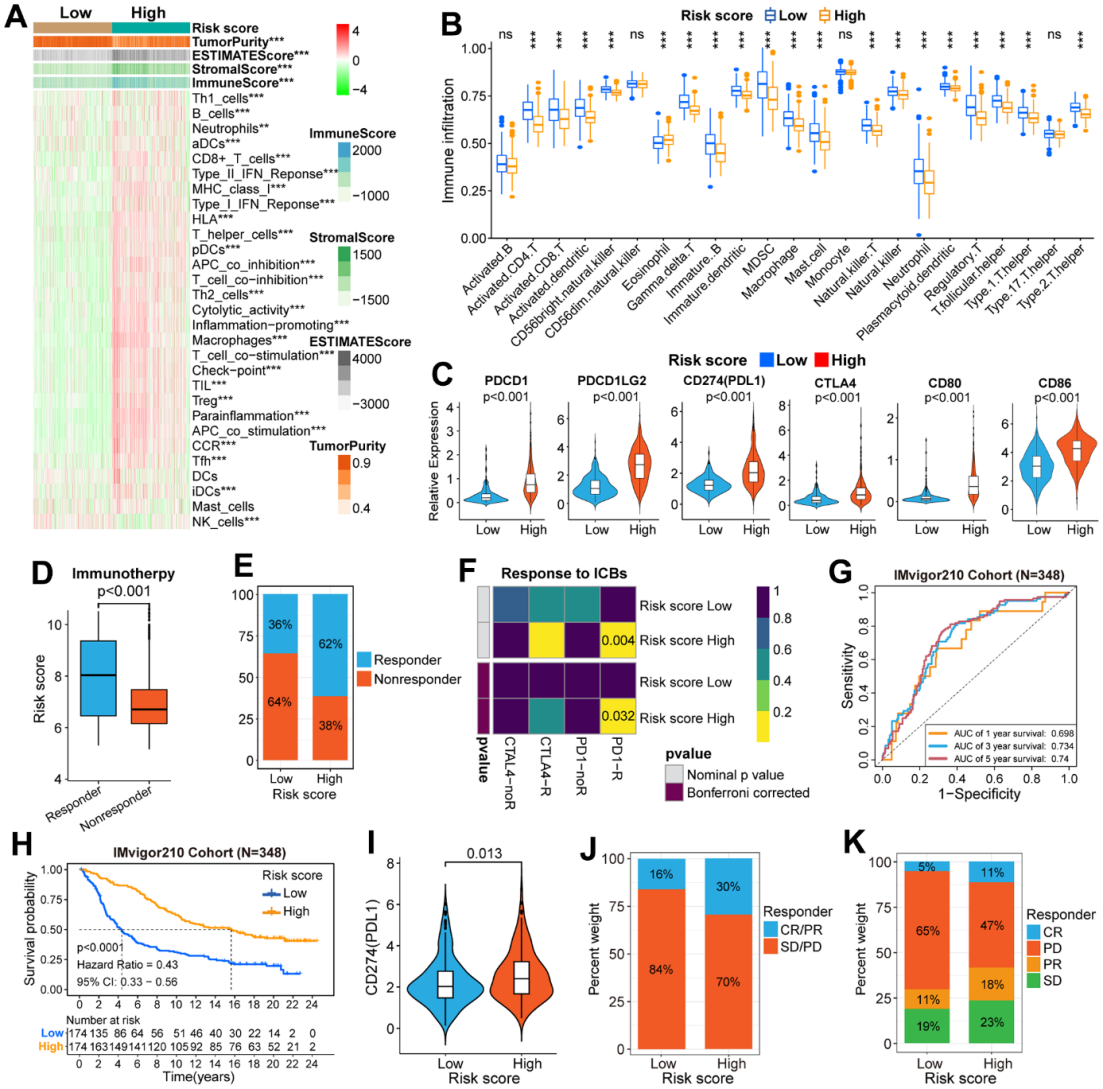
**Immune-related analysis in the TCGA and IMvigor210 cohorts.** (**A**) Difference analysis of immune signatures and four types of TME-related scores between high and low-risk groups in the TCGA cohort. (**B**) The difference in infiltration level of immune cells between high and low-risk groups. (**C**) Differential expression of six well-known ICPs between high and low-risk groups. (**D**) The difference in risk score between high and low-risk groups was stratified according to response to immunotherapy. (**E**) The difference in the distribution of responders and non-responders between the two risk groups. (**F**) The subgroup map predicted the response to ICB therapy between the two risk groups. (**G**) ROC curves verified the accuracy of the risk score in predicting the OS for patients in the IMvigor210 cohort. (**H**) The K-M survival curves of OS in patients between the high and low-risk groups. (**I**) Differential expression of CD274(PDL1) between high and low-risk groups. (**J**, **K**) Differences in the distribution of patients with and without response to ICI immunotherapy between high and low-risk groups. CR, complete response; PR, partial response; SD, stable disease; PD, progressive disease. CR/PR was identified as responders, and SD/PD was identified as non-responders. *P<0.05, **P<0.01, ***P<0.001, ****P<0.0001.

By performing GSVA in TCGA, CGGA, and GSE16011 cohorts, we found that the relative activities of 156 functional pathways were different between the two risk groups: the relative activity of 53 functional pathways increased in the low-risk group, mainly focusing on metabolic-related pathways, such as “butanoate metabolism”, “propanoate metabolism” and “glyoxylate and dicarboxylate metabolism”; the relative activity of other 103 functional pathways increased in the high-risk group, including immune-related pathways (Figure 5G and Supplementary Table 7). Next, we began an in-depth analysis of the relationship between immunologic characteristics and risk scores. We found that the TME-related scores were not only highly correlated with the risk score (Supplementary Figure 5C–5E) but also significantly different between the high and low-risk groups in the three cohorts (Figure 6A and Supplementary Figure 5A, 5B). Then, we also found that the enrichment scores of most immune signatures calculated by ssGSEA were different between the two risk groups in the three cohorts (Figure 6A and Supplementary Figure 5A, 5B). Considering that immune cells play a key role in tumorigenesis, tumor progression, and tumor immunity, we further explored the association between the scoring system and immune cells. The content of most immune cells was not only correlated with the risk score (Supplementary Figure 6A–6C) but also significantly different among different risk groups (Figure 6B and Supplementary Figure 6D, 6E). The results of the analysis between ICPs and risk scores (Figure 6C and Supplementary Figure 7A–7E) prompted us to continue to explore whether the scoring system can effectively predict the immunotherapy response of glioma patients. There was a significant difference of the risk score between the responder and non-responder groups (Figure 6D and Supplementary Figure 8A–8D). The proportion of responders in the high-risk group is much higher than those in the low-risk group, which indicates that glioma patients in the high-risk group are more likely to benefit from immunotherapy (Figure 6E and Supplementary Figure 8B–8E). The subclass mapping analysis indicated that glioma patients in the high-risk group are more sensitive to anti-PD-1 therapy (Bonferroni P = 0.016,0.032, 0.039 in GSE16011, TCGA, and CGGA cohorts, respectively) (Figure 6F and Supplementary Figure 8C–8F). If the Bonferroni corrected P > 0.05, the P-value would not be marked in the subgroup map, but from the color comparison of the corresponding patches in the subgroup map, it can be seen that the P-value of anti-CTLA4 therapy in the high-risk group is lower than that in the low-risk group, indicating that glioma patients in the high-risk group are more likely to respond to anti-CTLA4 therapy (Figure 6F and Supplementary Figure 8C–8F).

### Validation of the clinical application value of the scoring system in the IMvigor210 (mUC) cohort

We selected the IMvigor210 (mUC) cohort to further validate the clinical application value of the scoring system. K-M survival curves indicated that the OS of patients with a low-risk score was longer than those with a high-risk score (Figure 6H). Moreover, the risk score can effectively predict the 1, 3, and 5-year OS rates of patients in the IMvigor210 cohort (Figure 6G). Then, the differential expression of PDL1 (CD274) between high and low-risk groups was also explored. The results showed that the expression level of PDL1 in the high-risk group was higher than that in the low-risk group (Figure 6I). Furthermore, the proportion of CR/PR and PD/SD in the high-risk group is higher than that in the low-risk group (Figure 6J, 6K).

### COL1A2 is up-regulated in GBM

**Figure 7:**
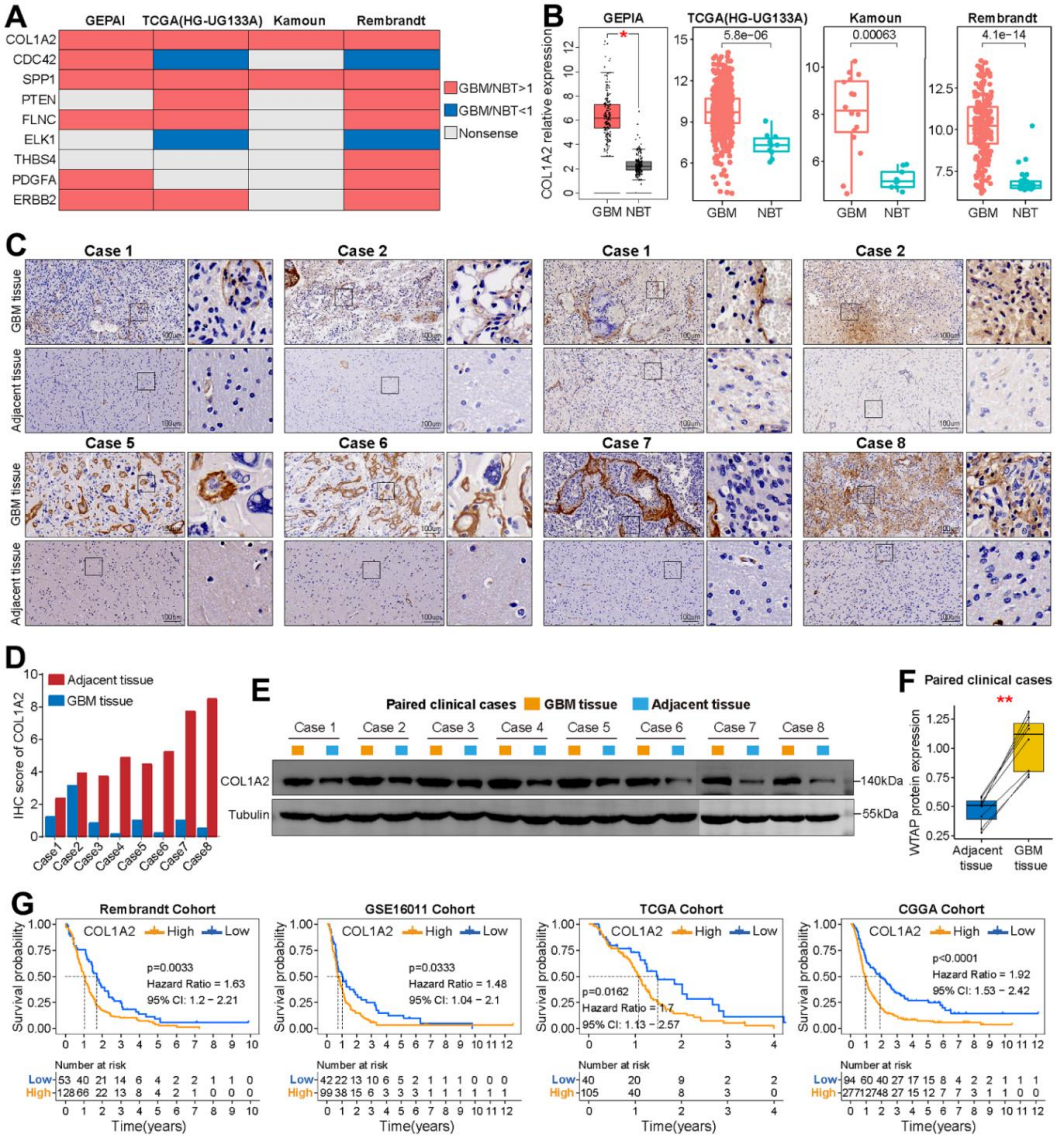
**Basic analysis of COL1A2.** (**A**) Summary of the differential expression analysis of nine FARGs in three independent GBM cohorts and the GEPIA website. Red represents up-regulated genes in GBM tissue, while blue represents a down-regulated gene in GBM tissue. Grey represents no statistical difference. (**B**) Differential expression analysis of COL1A2 between GBM tissues and NBTs in TCGA (HG-UG133A), Kamoun and Rembrandt cohorts, and GEPIA website. (**C**) Immunohistochemical analysis of COL1A2 protein in eight pairs of GBM tissues and adjacent nontumor tissues. (**D**) The IHC scores of COL1A2 in GBM tissues and matched adjacent nontumor tissues. (**E**, **F**) The protein expression level of COL1A2 in GBM tissues was significantly higher than that in corresponding adjacent tissues. (**G**) K-M survival curves of OS in GBM patients between the high and low COL1A2 expression groups. *P < 0.05, **P < 0.01.

First, compared with the other eight FARGs, the mRNA expression of COL1A2 in GBM tissues was significantly higher than that in normal brain tissues (NBT), which was verified on the GEPIA website and three independent GBM cohorts (Figure 7A, 7B). Then, immunohistochemical staining of eight pairs of GBM tissues and adjacent tissues showed that COL1A2 was significantly up-regulated in GBM tissues (Figure 7C, 7D). In addition, at the tissue protein level, the expression COL1A2 in GBM tissues was also significantly higher than that in corresponding adjacent tissues (Figure 7E, 7F). Finally, the K-M survival curves of COL1A2 in four independent GBM cohorts (Rembrandt, GSE16011, TCGA, and CGGA cohorts) showed that the prognosis outcomes of GBM patients in the high expression group were significantly shorter than that of GBM patients in the low expression group (Figure 7G).

### COL1A2 promotes the malignant progression of GBM cells *in vitro*

**Figure 8:**
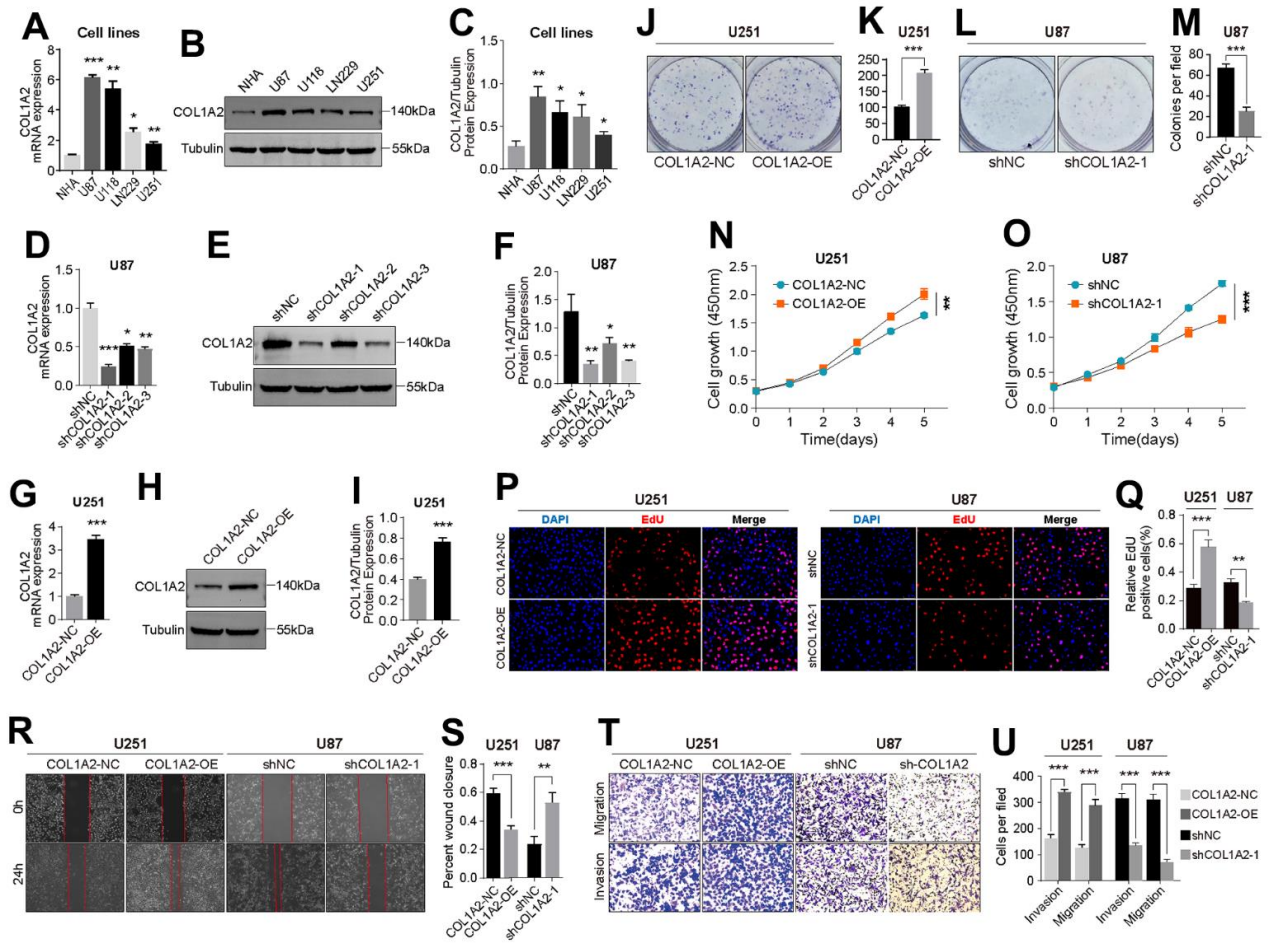
**COL1A2 promotes the proliferation, migration, and invasion of GBM cells *in vitro*.** (**A**–**C**) Differential expression of COL1A2 at transcriptional (**A**) and protein levels (**B**, **C**) between different GBM cell lines. (**D**–**F**) The efficiency of COL1A2 knock-down plasmid at transcriptional (**D**) and protein levels (**E**, **F**) was verified in U87 cells. (**G**–**I**) The efficiency of the COL1A2 overexpression plasmid at transcriptional (**G**) and protein levels (**H**, **I**) was verified in U251 cells. In (**A**–**I**), data are presented as the mean ± SD. X-axis: four different cell lines. Y-axis: expression level of genes. (**J**–**Q**) The proliferation activity of U251 and U87 cells were detected by colony formation assay (**J**–**M**), CCK-8 assay (**N**, **O**), and EdU staining (**P**, **Q**). (**R**–**U**) The migration and invasion ability of U87 and U251 cells were detected by wound healing (**R**, **S**) and Transwell assays (**T**, **U**). The representative photographs were photographed under a microscope. *P<0.05, **P<0.01, ***P<0.001.

By comparing the differential expression of COL1A2 between different cell lines at the transcription level and protein level, we found that the expression level of COL1A2 was the highest in U87 cells and the lowest in U251 cells (Figure 8A–8C). Then, we constructed knockdown and overexpression plasmids of COL1A2 to further explore the effect of COL1A2 on the biological behavior of GBM *in vitro*. The result of RT-qPCR and western blotting showed that the constructed knockdown and overexpression plasmids could significantly reduce or increase the expression of COL1A2 (Figure 8D–8I). The results of colony formation assay (Figure 8J–8M), CCK-8 assay (Figure 8N, 8O), and EdU staining (Figure 8P, 8Q) suggest that COL1A2 can significantly enhance the proliferation of GBM cells. In addition, the outcomes of wound healing and transwell assay support COL1A2 promoting the migration and invasion of GBM cells (Figure 8R–8U). These results suggest that COL1A2 plays an important role in the progression of GBM cells.

### Detection of the viability of GBM cells *in vitro*

**Figure 9:**
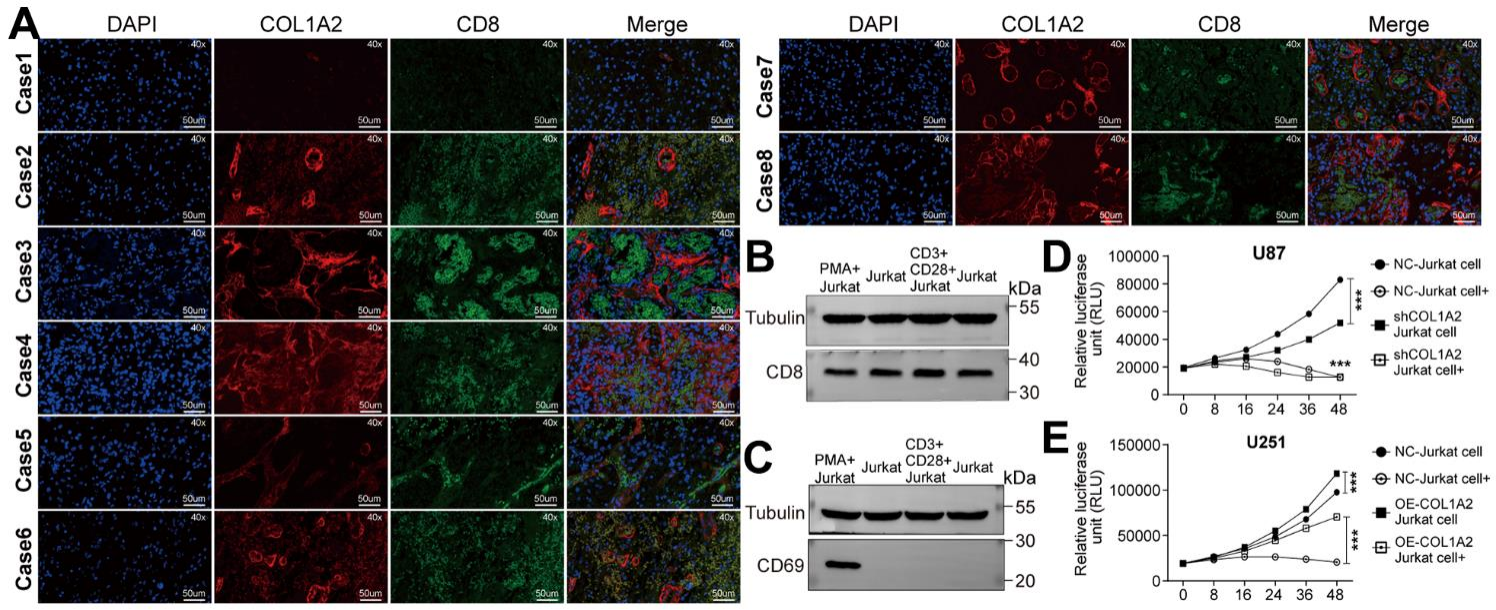
**COL1A2 inhibits the effect of activated Jurkat cells on the viability of GBM cells *in vitro*.** (**A**) Immunofluorescence staining of the nucleus, COL1A2, and CD8 in eight cases of GBM tissues. (**B**, **C**) Jurkat cells were stimulated by PMA and ionomycin, or by anti-CD3 and anti-CD28 antibodies. After 24 hours of stimulation, the expression of CD8 and CD69 proteins were detected. (**D**, **E**) The viability of U87 (**D**) and U251 (**E**) cells were analyzed by measuring luciferase activity. ***P<0.001.

In eight cases of GBM tissues, we found that the tissues with high expression of COL1A2 were accompanied by high expression of CD8. On the contrary, the tissues with low expression of COL1A2 were accompanied with low expression of CD8 (Figure 9A), which to some extent suggested that the expression of COL1A2 might induce the infiltration of CD8 T cells or the infiltration COL1A2. Then, we studied whether COL1A2 played a of CD8 T cells might induce the expression of role in the process of Jurkat cells acting on GBM cells *in vitro*. Both methods, PMA, and ionomycin or anti- CD3 and anti-CD28 antibodies, can effectively activate Jurkat cells into CD8 + Jurkat cells (Figure 9B). CD8 T cells transiently overexpress CD69 in the early stage of activation, which is considered to be a costimulatory signal of T cell proliferation. However, we did not detect the expression of CD69 protein in Jurkat cells stimulated by anti-CD3 and anti-CD28 antibodies (Figure 9C), so we utilized PMA and ionomycin to stimulate Jurkat cells. The results of the cell viability assay showed that activated Jurkat cells could effectively inhibit the viability of U87 (Figure 9D) and U251 (Figure 9E) cells. With the increase of COL1A2 expression, the lethality of activated Jurkat cells to GBM cells decreases, which may be related to COL1A2 promoting the proliferation of GBM cells.

## DISCUSSION

The prognosis of glioma patients remains poor. As such, it is critical to identify new prognostic biomarkers and develop a more effective treatment. Although there have been multiple studies on immunotherapy, especially by ICB in GBM patients, the results of phase III clinical trials have not been satisfactory compared to other tumors [29, 30]. Neither anti-CTLA-4 antibody alone nor in combination with anti-PD-1 antibody yielded long-term survival benefits for glioma patients [31]. Numerous factors can affect the response of glioma to immunotherapy including factors in the TME and TMB. The latter is a biomarker for evaluating the therapeutic effect of anti-PD-1 antibody [32]; and converting the immunosuppressive TME to a immunostimulatory one is an effective treatment strategy [33, 34]. In this study, we developed a novel molecular classification for glioma patients based on FARGs. Not only the OS and PFS outcomes of glioma patients are significantly different between different subtypes, but also the characteristics of TME are significantly different between different subtypes. However, we did not further validate the properties of the new molecular classification in multiple glioma cohorts, making it difficult to effectively validate its clinical application value. In addition, a scoring system was constructed based on nine FARGs (SPP1, THBS4, ERBB2, ELK1, COL1A2, PTEN, CDC42, FLNC, and PDGFA). The expression levels of these FARGs differed between different WHO grades. PTEN regulates signaling pathways related to cell growth and survival [35, 36] and cell metabolism [37]. COL1A2 participates in collagen synthesis [38] but has been implicated in the immune response [39]. ELK1 is a transcription factor that activates target genes via some protein kinase/regulatory kinase pathways [40, 41]. ERBB2 is a marker gene in breast cancer. SPP1 is known to be overexpressed in many malignant tumors including glioma [42–46] and regulates cell growth, proliferation, apoptosis, and migration [47]. THBS4 is a member of the thrombospondin protein family and plays important roles in wound healing and tissue repair [48–51], intracellular migration, adhesion, and proliferation [52–54]. FLNC is an action-binding filamin protein that regulates actin reorganization-dependent processes such as differentiation, migration, and proliferation of cells [55]. As a Rho family GTPase, CDC42 plays a key role in the activation of signaling cascades that regulate cell adhesion, cytoskeletal composition, proliferation, and migration and is therefore important for the malignant transformation of tumors [56]. In this study, COL1A2 was screened from nine FARGs used to construct the scoring system using bioinformatics analysis methods for further research, but no corresponding studies were conducted on the other eight FARGs, which will be the focus of our future research.

The clinical application value of the scoring system has been verified in TCGA, CGGA, and GSE16011 cohorts. The scoring system can not only effectively predict the prognosis outcome of glioma patients, but also can be used as an independent prognostic factor for glioma patients. TMB can predict the immunotherapeutic response of some types of tumors and has a close relationship with the scoring system. There are also significant differences in the distribution of mutation types of some classic genes between high and low-risk groups. Moreover, the TME between high and low-risk groups is also significantly different, which will directly affect the immunotherapeutic response of glioma patients. In addition to verifying the clinical application value of the scoring system in different glioma cohorts, we also found that the scoring system has an important clinical application value in the imvigor210 (mUC) cohort. Although we have conducted a comprehensive bioinformatics analysis of the scoring system, we have not conducted a comparative study on its clinical application value with other existing scoring systems, resulting in a lack of horizontal comparison.

To further study the role of FARGs in the progression of GBM, COL1A2 was screened and further studied. The screening process of COL1A2 is simple, and if it. can be screened from multiple perspectives, it would make the screening process more convincing. COL1A2 is upregulated in GBM tissue and promotes malignant progression of GBM cells, but its specific mechanism has not been thoroughly studied, which will be our future research focus. It has been suggested that the poor response to immunotherapy in glioma patients is attributable to immunosuppression in the brain [27], however, the specific mechanism is still unclear at present. COL1A2 was still taken as the research object. It was found that it may promote the infiltration of CD8 T cells, but at the same time may inhibit the lethality of CD8 T cells on tumor cells. As for the specific role of COL1A2 in the tumor microenvironment of glioma and in the immunotherapy process, if further research can be made in this study, it would make this study more valuable in clinical application.

In summary, we identified two distinct subtypes based on FARGs. More importantly, we established a scoring system with great clinical application value, which can not only effectively predict the prognosis outcome of glioma patients, but also predict the immunotherapy response for glioma patients. In addition, we screened COL1A2 and verified its involvement in the progression of GBM cells *in vitro*. It is expected that this study can contribute to the diagnosis and treatment of glioma patients.

## MATERIALS AND METHODS

### Data sources

The flow diagram of our study was shown in Figure 1. RNA-seq data, somatic mutation data, and corresponding clinical annotation of glioma patients were downloaded from the TCGA database, CGGA database, GEO database, UCSC Xena portal, GlioVis website, and NIH website. Before comparing and analyzing the gene expression data from different platforms, we performed transcripts per kilobase million (TPM) values transformation and robust multichip averaging (RMA) values transformation on RNA-seq data and microarray data, respectively. Patients with incomplete clinical information (including OS, OS status, gender, age, and WHO grade) will not be included in the clinical correlation analysis. Demographics and clinical information of 1813 glioma patients are shown in Supplementary Table 1. Somatic mutation data were processed with the R package “maftools” [57]. The genomic identification algorithm of important targets in tumors is used to process CNV data [58]. We searched 199 FARGs from the Molecular Signatures Database and the details of these FARGs can be found in Supplementary Table 2. In addition, the IMvigor210 cohort treated with the PD-L1 inhibitor was also included in this study to confirm our findings.

### Acquisition of GBM tissue samples

The current research was authorized by the Ethics Committee of The First People’s Hospital of Fuzhou City and informed consent was obtained from the patients. The surgical specimens of GBM patients were removed to liquid nitrogen immediately after surgical excision.

### Unsupervised consensus clustering based on FARGs

Different glioma cohorts have different sequencing methods and gene annotation information. To facilitate follow-up analysis, we took the intersection of 199 FARGs in three independent glioma cohorts and finally obtained 192 FARGs. A novel molecular classification was identified based on the 192 FARGs extracted from the TCGA cohort by unsupervised consensus clustering with 1000 iterations using the “ConsensusClusterPlus” package [59]. The optimal value of molecular classification should consider not only the rate of increase of the area under the cumulative distribution function (CDF) curves but also consider the correlation between different molecular classifications.

### Gene set variation analysis (GSVA) between different subtypes

After downloading “c2.cp.kegg.v7.2.symbols” from the Molecular Signatures Database, GSVA between different subtypes was performed using the R package “GSVA” [60]. Differences in the relative activity of functional pathways between different subtypes were explored by using the R package “limma” [61].

### Immune-related analysis between different subtypes

Different types of cell components in tumor tissues can be estimated by calculating TME-related scores using the R package “ESTIMATE” [62]. The content of different types of immune cells was reckoned using MCPCOUNTER, CIBERSORT, and QUANTISEQ algorithms [63, 64]. Enrichment scores of 29 immune signatures were determined by using a single sample gene set enrichment analysis (ssGSEA), which to some extent represents the immune activity within the tumor. [65]. The analyzed immune checkpoint proteins (ICPs) were selected from a previous study [66]. The above immune-related characteristics were analyzed in different subtypes.

### Construction of a scoring system and verification of its prognostic value

We screened 175 FARGs with prognostic significance from 192 FARGs using uni-Cox regression analysis. 175 FARGs were further screened by lasso algorithm and multi-Cox regression analysis, and nine FARGs were finally obtained to construct a scoring system: risk score = sum (gene expression × coefficient). High and low-risk groups were classified according to the median value of the risk score. The prognostic value of the scoring system was evaluated by using Kaplan–Meier (K–M) survival curves (R package “survminer”), receiver operating characteristic (ROC) curves (R package “survivalROC”), uni- and multi-Cox regression analysis (R package “survival” and “forestplot”). The different distribution of different clinicopathological characteristics between high and low-risk groups was analyzed by R package “limma”.

### Genomic variation analysis between high and low-risk groups

The mutation type and frequency of genes between high and low-risk groups were analyzed by the R packages “maftool” [57] and “GenVisR” [67]. The relationship between TMB, as a marker for the therapeutic efficacy of anti-PD-1 antibodies in other cancers [32, 68], and risk score was also analyzed by R package “ggpubr”. As genomic alterations can impact tumor immunity and immune infiltration patterns [69, 70], we compared amplifications and deletions between high and low-risk groups and visualized the results as circle graphs using the R package “RCircos” [71].

### Immune-related analysis of risk score

At present, only a sub-fraction of patients achieved long-lasting clinical benefits from ICBs treatment. To effectively predict the response of tumor patients to ICBs, the TIDE algorithm was developed mainly to model two primary mechanisms of tumor immune evasion: inducing the dysfunction of cytotoxic T lymphocytes (CTLs) in tumors and preventing the infiltration of CTLs in tumor tissues [72]. T cell dysfunction was identified by measuring the interaction between each gene and the infiltration level of CTL to influence patient survival. The TIDE algorithm explored the association between gene expression data and markers of T cell dysfunction. Tumor samples that were highly positively correlated with markers of T cell dysfunction were identified as non-responders and otherwise as responders. Finally, the TIDE algorithm and an unsupervised subclass mapping method [73] were used together to forecast the response of glioma patients to anti–PD-1 and anti–CTLA-4 immunotherapy.

### Cell culture, transfection, and activation

Normal human astrocytes (NHA) and GBM cell lines (U251, LN229, U118, and U87) were obtained from the Shanghai Institute of Biosciences and Cell Resources Center. Jurkat cells were purchased from The Global Bioresource Center. Except that Jurkat cells were cultured with RPMI-1640 medium (Gibco, USA), other cell lines were cultured with MEM (Gibco, USA) or DMEM (Gibco, USA). 50 IU/ml penicillin, 50 μg/ml streptomycin, and fetal bovine serum (FBS, Gibco, USA) were added to the culture medium. The COL1A2 knockdown and overexpression plasmids were constructed by the Sheweisi Biotechnology Company (Tianjin, China). U6-MCS-SV40 polyA-CMV-EGFP-SV40-NeoR was used to construct COL1A2 overexpression plasmid, and its three target sequences are shCOL1A2-1, 5’-GGTGTAAGCGGTGGTGGTTAT-3’, shCOL1A2-2, 5’-GCACTATGGATGCTATCAAAG-3’ and shCOL1A2-3, 5’-GCAACAGCAGGTTCACTTACA-3’ respectively. CMV-MCS-EF1a-ZsGreen1-SV40-Neomycin was used to construct COL1A2 overexpression plasmid, and its upstream and downstream primers are “AGCTGGCTAGCGTTCTCGAGGCCACCATGCTCAGCTTTGTGGATACGC” and “GTCTTTTTATTGCCGGGTACCTTATTTGAAACAGACTGGGCCAATG” respectively. Lipofectamine 3000 (Thermo Fisher, L3000075, USA) was used to transfect plasmids when the cell adhesion density reached about 80%. Two methods were used to activate Jurkat cells. Method 1: Jurkat cells (1 × 10^6^/ml) were stimulated by adding 20 ng/ml phorbol 12-myristate 13-acetate (PMA, Acmec, P33390-1mg) and 500 ng/ml ionomycin (70-CS0002, MultiSciences, China). Method 2: Jurkat cells were stimulated by adding 5μg/ml of anti-CD3 antibody (17617-1-AP, Proteintech, China), which was coated in a 6-well plate with the 5μg/ml of anti-CD28 antibody (65099-1-Ig, Proteintech). The expression of CD8 protein was used to detect whether Jurkat cells were activated, and the expression of CD69 protein was used to detect whether Jurkat cells proliferated. Jurkat and GBM cells were co-cultured (Jurkat: GBM cells = 1:1) in 96-well plates with a total volume of 200 μl.

### RT-qPCR and Western blotting

The primer sequences of COL1A2 and GAPDH (internal reference gene) purchased from Ribobio (Guangzhou, China) are as follows: COL1A2 F primer: 5’-GGCCCTCAAGGTTTCCAAGG-3’, R primer: 5’-CACCCTGTGGTCCAACAACTC-3’; GAPDH F primer: 5’-TGTGGGCATCAATGGATTTGG-3’, R primer: 5’-ACACCATGTATTCCGGGTCAAT-3’. The primary antibodies include anti-COL1A2 (Servicebio, GB13022-2, 1:3000 dilution), anti-CD8 (Proteintech, 66868-1-Ig, 1:4000 dilution), anti-CD69 (Proteintech, 10803-1-AP, 1:1500 dilution) and anti-β tubulin (Proteintech, 11224-1-AP, 1:8000 dilution). The secondary antibodies included goat anti-rabbit IgG (Abcam, ab6721 1:5000 dilution) and goat anti-mouse IgG (Abcam, ab6789 1:10000 dilution). The extraction and detection methods of protein and total RNA in different cell lines were completely consistent with those described in our previous study [74]. Here, we will describe the extraction steps of tissue protein in detail. First, the tissues were weighed and sheared into 2ml EP tubes. RIPA and PMSF were mixed in a ratio of 100:1 to prepare tissue lysates. 1ml of lysate was added to every 50mg of sheared tissue and homogenized with a homogenizer, and then lysed on ice for 30 minutes. Finally, the mixture of lysate and tissue was centrifuged with a low-temperature high-speed centrifuge at 4° C and 12000 rpm for 10 minutes. The subsequent steps are the same as the extraction of cellular proteins. Both RT-qPCR and western blotting assays were repeated three times.

### Immunohistochemical and immunofluorescence staining

First, eight pairs of GBM and adjacent tissues were fixed with 10% formalin for seven days, and then these tissues were embedded with paraffin and sectioned. After dewaxing and dehydration, the tissue sections were treated with 3% hydrogen peroxide for 10 minutes. Subsequently, the tissue sections were blocked with 5% BSA and then incubated overnight with a primary antibody against COL1A2 (Servicebio, GB13022-2, 1:1000 dilution) at 4° C. Then the tissue sections were treated with the corresponding secondary antibodies at room temperature for one hour. Finally, DAB staining, target molecule detection, and hematoxylin re-staining were carried out in turn. For immunofluorescence staining, tissue sections were immune-stained overnight with primary antibodies against COL1A2 (Servicebio, GB13022-2, 1:1000 dilution) and CD8 (Proteintech, 1:400) at 4° C, and then incubated with fluorochrome-conjugated antibodies. DAPI was added as a nuclear counterstain.

### Cell proliferation assay

Cells were seeded into 6-well plates at 1000 cells/well and cultured for about 15 days. It was predicted that the cells would proliferate for about 5-7 generations. The culture medium was changed every three days, and the colony formation was closely observed. Cell culture was stopped when the number of cells in a single colony approached 50. The colonies were fixed with 4% ice-precooled paraformaldehyde for about 20 minutes, then stained with 0.1% crystal violet for about 20 minutes.

The Cell Counting Kit-8 (CCK-8, Beyotime, Shanghai, China) was employed to assay cell proliferation activity. Cells were seeded into 96-well plates at 2000 cells/well. Adding 10ul CCK-8 reagent to each well and continuing to culture in the incubator for two hours, the absorbance of cells was measured at 450nm every 24 hours for 5 consecutive days.

2 × 104 cells were seeded into each well of the 24-well plate and cultured until the cell adhesion concentration reached about 75%. BeyoClick™ EdU Cell Proliferation Kit (Beyotime, Shanghai, China) was used to measure cell proliferation activity according to the manufacturer’s instructions. The nuclei of all cells with blue fluorescence and the positive cells with red fluorescence were photographed by fluorescence microscopy, and the results were analyzed by ImageJ software.

### Cell migration and invasion assay

When the adhesion concentration of U251 and U87 cells in the six-well plates reached 85-90%, the tip of a 200μl sterile spear cuts through the cell layer at the bottom of the plate to form an artificial wound. After washing the non-adherent cells with phosphate-buffered saline (PBS), the adherent cells were further cultured in a serum-free medium. At 0 and 24 hours, wound closure was photographed using an inverted Leica microscope.

Transwell chambers (Corning, USA) were utilized to assay the invasion and migration of GBM cell lines. First, the Matrigel (Corning, 356234, USA) with a concentration of 500 μg/ml was used to cover the upper chambers. 200μl serum-free medium and 600μl medium containing 10% FBS were added to the upper chamber and the lower chamber, respectively, and 8×10^4^ transfected cells were seeded in the upper chamber. After 24 hours of incubation at 37° C and 5% CO_2_ for 24 hours, the non-invasive cells on the upper chambers were removed with a microcell scraper. The following operations were carried out at room temperature. The cells at the bottom of the upper chamber were fixed with 4% ice-precooled formaldehyde (Solarbio, P1110, China) for 30 minutes and then washed twice with PBS, and then the chambers were placed in 0.1% crystal violet (Solarbio, G1075, China) for 20 minutes. Finally, the chambers were washed with PBS, dried, and photographed with a microscope (Leica Microsystems, D-35578). In addition, we also performed the migration assay in the same way as the invasion assay, except that the upper chamber was not covered with Matrigel. Both invasion and migration assays were repeated three times.

### Tumor cell viability assay

In light of the instructions offered by the manufacturer, the lentivirus expressing luciferase designed by Sheweisi Biotechnology Company (Tianjin, China) was transfected into U251 and U87 cells and screened with neomycin. Then COL1A2 knockdown and overexpression plasmids were transfected into cells stably expressing luciferase, respectively. When these cells grew to about 90%, they have inoculated with Jurkat cells in a ratio of 1:1 to 96 well plates with a white and clear bottom (total 1.0×10^4^ cells/ml). Co-cultured cells were taken at different time points (8, 16, 24, 36, 48 h), the supernatant was discarded, washed with PBS, cell lysate was added, and then D-luciferin potassium salt (D1009, UElandy, China) was added. After the above operations are completed, the fluorescence intensity is detected using a multifunctional microplate reader. The experiment was repeated three times.

### Statistical analyses

Spearman and Pearson analysis methods were used to determine correlations between different grouping variables. The unpaired Student’s t-test and Mann–Whitney U-test were used to analyzing normally and non-normally distributed data, respectively. The Wilcoxon rank-sum test was used to compare two groups or categories; for more than two groups or categories, the Kruskal–Wallis test was used. Survival was assessed by K–M analysis with the log-rank test. Cox regression analyses were carried out to evaluate the prognostic value and stability of the risk prediction model. ROC curves were used to assess the prognostic value of 1-, 3-, and 5-year OS. TIIC infiltration level was analyzed using three algorithms (CIBERSORT, QUANTISEQ, and MCPCOUNTER). All statistical tests were two-sided and *P*<0.05 was considered statistically significant.

### Availability of data and materials

The Cancer Genome Atlas (TCGA) database: https://portal.gdc.cancer.gov/. Chinese Glioma Genome Atlas (CGGA) database: http://www.cgga.org.cn/. Gene Expression Omnibus (GEO) databse: https://www.ncbi.nlm.nih.gov/geo/. National Human Genome Research Institute Home (NIH): https://www.genome.gov/. Data Visualization Tools for Brain Tumor Datasets (GlioVis): http://gliovis.bioinfo.cnio.es/. Molecular Signatures Database: https://www.gsea-msigdb.org/gsea/index.jsp. Gene Expression Profiling Interactive Analysis (GEPIA) database: http://gepia.cancer-pku.cn/detail.php. Tumor Immune Dysfunction and Exclusion (TIDE): http://tide.dfci.harvard.edu/. IMvigor210 cohort: http://research-pub.gene.com/IMvigor210CoreBiologies).

## Supplementary Material

Supplementary Figures

Supplementary Table 1

Supplementary Table 2

Supplementary Table 3

Supplementary Table 4

Supplementary Table 5

Supplementary Table 6

Supplementary Table 7

## References

[1] Ostrom QT, Gittleman H, Stetson L, Virk SM, Barnholtz-Sloan JS. Epidemiology of gliomas. Cancer Treat Res. 2015; 163:1–14. 10.1007/978-3-319-12048-5_1 25468222

[2] Jiang T, Nam DH, Ram Z, Poon WS, Wang J, Boldbaatar D, Mao Y, Ma W, Mao Q, You Y, Jiang C, Yang X, Kang C, et al, and Chinese Glioma Cooperative Group (CGCG), and Society for Neuro-Oncology of China (SNO-China), and Chinese Brain Cancer Association (CBCA), and Chinese Glioma Genome Atlas (CGGA), and Asian Glioma Genome Atlas (AGGA) network. Clinical practice guidelines for the management of adult diffuse gliomas. Cancer Lett. 2021; 499:60–72. 10.1016/j.canlet.2020.10.050 33166616

[3] Jhaveri J, Liu Y, Chowdhary M, Buchwald ZS, Gillespie TW, Olson JJ, Voloschin AD, Eaton BR, Shu HG, Crocker IR, Curran WJ, Patel KR. Is less more? Comparing chemotherapy alone with chemotherapy and radiation for high-risk grade 2 glioma: An analysis of the National Cancer Data Base. Cancer. 2018; 124:1169–78. 10.1002/cncr.31158 29205287

[4] Xu S, Tang L, Li X, Fan F, Liu Z. Immunotherapy for glioma: Current management and future application. Cancer Lett. 2020; 476:1–12. 10.1016/j.canlet.2020.02.002 32044356

[5] Stupp R, Taillibert S, Kanner A, Read W, Steinberg D, Lhermitte B, Toms S, Idbaih A, Ahluwalia MS, Fink K, Di Meco F, Lieberman F, Zhu JJ, et al. Effect of Tumor-Treating Fields Plus Maintenance Temozolomide vs Maintenance Temozolomide Alone on Survival in Patients With Glioblastoma: A Randomized Clinical Trial. JAMA. 2017; 318:2306–16. 10.1001/jama.2017.18718 29260225 PMC5820703

[6] Li BO, Meng C, Zhang X, Cong D, Gao X, Gao W, Ju D, Hu S. Effect of photodynamic therapy combined with torasemide on the expression of matrix metalloproteinase 2 and sodium-potassium-chloride cotransporter 1 in rat peritumoral edema and glioma. Oncol Lett. 2016; 11:2084–90. 10.3892/ol.2016.4210 26998126 PMC4774439

[7] Zeng T, Cui D, Gao L. Glioma: an overview of current classifications, characteristics, molecular biology and target therapies. Front Biosci (Landmark Ed). 2015; 20:1104–15. 10.2741/4362 25961548

[8] Gao HX, Wang MB, Li SJ, Niu J, Xue J, Li J, Li XX. Identification of Hub Genes and Key Pathways Associated with Peripheral T-cell Lymphoma. Curr Med Sci. 2020; 40:885–99. 10.1007/s11596-020-2250-9 32980897

[9] Lan Q, Wang P, Tian S, Dong W. Mining TCGA database for genes of prognostic value in gastric cancer microenvironment. J Cell Mol Med. 2020; 24:11120–32. 10.1111/jcmm.15595 32818296 PMC7576220

[10] Wehrle-Haller B. Structure and function of focal adhesions. Curr Opin Cell Biol. 2012; 24:116–24. 10.1016/j.ceb.2011.11.001 22138388

[11] Hong R, Gu J, Niu G, Hu Z, Zhang X, Song T, Han S, Hong L, Ke C. PRELP has prognostic value and regulates cell proliferation and migration in hepatocellular carcinoma. J Cancer. 2020; 11:6376–89. 10.7150/jca.46309 33033521 PMC7532499

[12] Li J, Hao N, Han J, Zhang M, Li X, Yang N. ZKSCAN3 drives tumor metastasis via integrin β4/FAK/AKT mediated epithelial-mesenchymal transition in hepatocellular carcinoma. Cancer Cell Int. 2020; 20:216. 10.1186/s12935-020-01307-7 32518525 PMC7275473

[13] Atallah J, Khachfe HH, Berro J, Assi HI. The use of heparin and heparin-like molecules in cancer treatment: a review. Cancer Treat Res Commun. 2020; 24:100192. 10.1016/j.ctarc.2020.100192 32673846

[14] Pitt JM, Marabelle A, Eggermont A, Soria JC, Kroemer G, Zitvogel L. Targeting the tumor microenvironment: removing obstruction to anticancer immune responses and immunotherapy. Ann Oncol. 2016; 27:1482–92. 10.1093/annonc/mdw168 27069014

[15] Eke I, Cordes N. Focal adhesion signaling and therapy resistance in cancer. Semin Cancer Biol. 2015; 31:65–75. 10.1016/j.semcancer.2014.07.009 25117005

[16] Correia AL, Bissell MJ. The tumor microenvironment is a dominant force in multidrug resistance. Drug Resist Updat. 2012; 15:39–49. 10.1016/j.drup.2012.01.006 22335920 PMC3658318

[17] Klemm F, Joyce JA. Microenvironmental regulation of therapeutic response in cancer. Trends Cell Biol. 2015; 25:198–213. 10.1016/j.tcb.2014.11.006 25540894 PMC5424264

[18] Nikou S, Arbi M, Dimitrakopoulos FD, Sirinian C, Chadla P, Pappa I, Ntaliarda G, Stathopoulos GT, Papadaki H, Zolota V, Lygerou Z, Kalofonos HP, Bravou V. Integrin-linked kinase (ILK) regulates KRAS, IPP complex and Ras suppressor-1 (RSU1) promoting lung adenocarcinoma progression and poor survival. J Mol Histol. 2020; 51:385–400. 10.1007/s10735-020-09888-3 32592097

[19] Pallasch FB, Schumacher U. Angiotensin Inhibition, TGF-β and EMT in Cancer. Cancers (Basel). 2020; 12:2785. 10.3390/cancers12102785 32998363 PMC7601465

[20] Fousek K, Horn LA, Palena C. Interleukin-8: A chemokine at the intersection of cancer plasticity, angiogenesis, and immune suppression. Pharmacol Ther. 2021; 219:107692. 10.1016/j.pharmthera.2020.107692 32980444 PMC8344087

[21] Landeros N, Santoro PM, Carrasco-Avino G, Corvalan AH. Competing Endogenous RNA Networks in the Epithelial to Mesenchymal Transition in Diffuse-Type of Gastric Cancer. Cancers (Basel). 2020; 12:2741. 10.3390/cancers12102741 32987716 PMC7598708

[22] Graeber MB, Scheithauer BW, Kreutzberg GW. Microglia in brain tumors. Glia. 2002; 40:252–9. 10.1002/glia.10147 12379912

[23] Rizvi NA, Hellmann MD, Snyder A, Kvistborg P, Makarov V, Havel JJ, Lee W, Yuan J, Wong P, Ho TS, Miller ML, Rekhtman N, Moreira AL, et al. Cancer immunology. Mutational landscape determines sensitivity to PD-1 blockade in non-small cell lung cancer. Science. 2015; 348:124–8. 10.1126/science.aaa1348 25765070 PMC4993154

[24] Neoadjuvant PD-1 Blockade in Resectable Lung Cancer; Nivolumab and Ipilimumab in Advanced Melanoma; Overall Survival with Combined Nivolumab and Ipilimumab in Advanced Melanoma; Prolonged Survival in Stage III Melanoma with Ipilimumab Adjuvant Therapy; Combined Nivolumab and Ipilimumab or Monotherapy in Untreated Melanoma; Combined Nivolumab and Ipilimumab or Monotherapy in Untreated Melanoma; Nivolumab and Ipilimumab versus Ipilimumab in Untreated Melanoma; Rapid Eradication of a Bulky Melanoma Mass with One Dose of Immunotherapy; Genetic Basis for Clinical Response to CTLA-4 Blockade; Genetic Basis for Clinical Response to CTLA-4 Blockade in Melanoma; Nivolumab plus Ipilimumab in Advanced Melanoma; Safety and Tumor Responses with Lambrolizumab (Anti-PD-1) in Melanoma; Hepatotoxicity with Combination of Vemurafenib and Ipilimumab. N Engl J Med. 2018; 379:2185. 10.1056/NEJMx180040 31442371

[25] Topalian SL, Hodi FS, Brahmer JR, Gettinger SN, Smith DC, McDermott DF, Powderly JD, Carvajal RD, Sosman JA, Atkins MB, Leming PD, Spigel DR, Antonia SJ, et al. Safety, activity, and immune correlates of anti-PD-1 antibody in cancer. N Engl J Med. 2012; 366:2443–54. 10.1056/NEJMoa1200690 22658127 PMC3544539

[26] Yang T, Kong Z, Ma W. PD-1/PD-L1 immune checkpoint inhibitors in glioblastoma: clinical studies, challenges and potential. Hum Vaccin Immunother. 2021; 17:546–53. 10.1080/21645515.2020.1782692 32643507 PMC7899692

[27] Touat M, Li YY, Boynton AN, Spurr LF, Iorgulescu JB, Bohrson CL, Cortes-Ciriano I, Birzu C, Geduldig JE, Pelton K, Lim-Fat MJ, Pal S, Ferrer-Luna R, et al. Mechanisms and therapeutic implications of hypermutation in gliomas. Nature. 2020; 580:517–23. 10.1038/s41586-020-2209-9 32322066 PMC8235024

[28] Daubon T, Hemadou A, Romero Garmendia I, Saleh M. Glioblastoma Immune Landscape and the Potential of New Immunotherapies. Front Immunol. 2020; 11:585616. 10.3389/fimmu.2020.585616 33154756 PMC7591769

[29] Reardon DA, Brandes AA, Omuro A, Mulholland P, Lim M, Wick A, Baehring J, Ahluwalia MS, Roth P, Bähr O, Phuphanich S, Sepulveda JM, De Souza P, et al. Effect of Nivolumab vs Bevacizumab in Patients With Recurrent Glioblastoma: The CheckMate 143 Phase 3 Randomized Clinical Trial. JAMA Oncol. 2020; 6:1003–10. 10.1001/jamaoncol.2020.1024 32437507 PMC7243167

[30] Weller M, Butowski N, Tran DD, Recht LD, Lim M, Hirte H, Ashby L, Mechtler L, Goldlust SA, Iwamoto F, Drappatz J, O’Rourke DM, Wong M, et al, and ACT IV trial investigators. Rindopepimut with temozolomide for patients with newly diagnosed, EGFRvIII-expressing glioblastoma (ACT IV): a randomised, double-blind, international phase 3 trial. Lancet Oncol. 2017; 18:1373–85. 10.1016/S1470-2045(17)30517-X 28844499

[31] Reardon DA, Gokhale PC, Klein SR, Ligon KL, Rodig SJ, Ramkissoon SH, Jones KL, Conway AS, Liao X, Zhou J, Wen PY, Van Den Abbeele AD, Hodi FS, et al. Glioblastoma Eradication Following Immune Checkpoint Blockade in an Orthotopic, Immunocompetent Model. Cancer Immunol Res. 2016; 4:124–35. 10.1158/2326-6066.CIR-15-0151 26546453

[32] Le DT, Durham JN, Smith KN, Wang H, Bartlett BR, Aulakh LK, Lu S, Kemberling H, Wilt C, Luber BS, Wong F, Azad NS, Rucki AA, et al. Mismatch repair deficiency predicts response of solid tumors to PD-1 blockade. Science. 2017; 357:409–13. 10.1126/science.aan6733 28596308 PMC5576142

[33] Duan Q, Zhang H, Zheng J, Zhang L. Turning Cold into Hot: Firing up the Tumor Microenvironment. Trends Cancer. 2020; 6:605–18. 10.1016/j.trecan.2020.02.022 32610070

[34] Zhang Z, Bao S, Yan C, Hou P, Zhou M, Sun J. Computational principles and practice for decoding immune contexture in the tumor microenvironment. Brief Bioinform. 2021; 22:bbaa075. 10.1093/bib/bbaa075 32496512

[35] Stiles B, Groszer M, Wang S, Jiao J, Wu H. PTENless means more. Dev Biol. 2004; 273:175–84. 10.1016/j.ydbio.2004.06.008 15328005

[36] Downes CP, Ross S, Maccario H, Perera N, Davidson L, Leslie NR. Stimulation of PI 3-kinase signaling via inhibition of the tumor suppressor phosphatase, PTEN. Adv Enzyme Regul. 2007; 47:184–94. 10.1016/j.advenzreg.2006.12.018 17343901

[37] Stiles BL. Phosphatase and tensin homologue deleted on chromosome 10: extending its PTENtacles. Int J Biochem Cell Biol. 2009; 41:757–61. 10.1016/j.biocel.2008.09.022 18950730 PMC2940266

[38] Liu J, Luo C, Yin Z, Li P, Wang S, Chen J, He Q, Zhou J. Downregulation of let-7b promotes COL1A1 and COL1A2 expression in dermis and skin fibroblasts during heat wound repair. Mol Med Rep. 2016; 13:2683–8. 10.3892/mmr.2016.4877 26861712

[39] Tao R, Fan XX, Yu HJ, Ai G, Zhang HY, Kong HY, Song QQ, Huang Y, Huang JQ, Ning Q. MicroRNA-29b-3p prevents Schistosoma japonicum-induced liver fibrosis by targeting COL1A1 and COL3A1. J Cell Biochem. 2018; 119:3199–209. 10.1002/jcb.26475 29091295

[40] Hipskind RA, Rao VN, Mueller CG, Reddy ES, Nordheim A. Ets-related protein Elk-1 is homologous to the c-fos regulatory factor p62TCF. Nature. 1991; 354:531–4. 10.1038/354531a0 1722028

[41] Gille H, Kortenjann M, Thomae O, Moomaw C, Slaughter C, Cobb MH, Shaw PE. ERK phosphorylation potentiates Elk-1-mediated ternary complex formation and transactivation. EMBO J. 1995; 14:951–62. 10.1002/j.1460-2075.1995.tb07076.x 7889942 PMC398167

[42] Overgaard J, Eriksen JG, Nordsmark M, Alsner J, Horsman MR, and Danish Head and Neck Cancer Study Group. Plasma osteopontin, hypoxia, and response to the hypoxia sensitiser nimorazole in radiotherapy of head and neck cancer: results from the DAHANCA 5 randomised double-blind placebo-controlled trial. Lancet Oncol. 2005; 6:757–64. 10.1016/S1470-2045(05)70292-8 16198981

[43] Hu H, Liu Z, Liu C. Correlation of OPN gene expression with proliferation and apoptosis of ovarian cancer cells and prognosis of patients. Oncol Lett. 2019; 17:2788–94. 10.3892/ol.2019.9896 30854053 PMC6365894

[44] Cho H, Hong SW, Oh YJ, Kim MA, Kang ES, Lee JM, Kim SW, Kim SH, Kim JH, Kim YT, Lee K. Clinical significance of osteopontin expression in cervical cancer. J Cancer Res Clin Oncol. 2008; 134:909–17. 10.1007/s00432-007-0351-5 18210151 PMC12160727

[45] Kovacheva M, Zepp M, Schraad M, Berger S, Berger MR. Conditional Knockdown of Osteopontin Inhibits Breast Cancer Skeletal Metastasis. Int J Mol Sci. 2019; 20:4918. 10.3390/ijms20194918 31590218 PMC6801824

[46] Güttler A, Giebler M, Cuno P, Wichmann H, Keßler J, Ostheimer C, Söling A, Strauss C, Illert J, Kappler M, Vordermark D, Bache M. Osteopontin and splice variant expression level in human malignant glioma: radiobiologic effects and prognosis after radiotherapy. Radiother Oncol. 2013; 108:535–40. 10.1016/j.radonc.2013.06.036 23891093

[47] Qin H, Wang R, Wei G, Wang H, Pan G, Hu R, Wei Y, Tang R, Wang J. Overexpression of osteopontin promotes cell proliferation and migration in human nasopharyngeal carcinoma and is associated with poor prognosis. Eur Arch Otorhinolaryngol. 2018; 275:525–34. 10.1007/s00405-017-4827-x 29214433

[48] Adams JC. Thrombospondins: multifunctional regulators of cell interactions. Annu Rev Cell Dev Biol. 2001; 17:25–51. 10.1146/annurev.cellbio.17.1.25 11687483

[49] Adams JC, Lawler J. The thrombospondins. Int J Biochem Cell Biol. 2004; 36:961–8. 10.1016/j.biocel.2004.01.004 15094109 PMC2885884

[50] Bornstein P. Thrombospondins as matricellular modulators of cell function. J Clin Invest. 2001; 107:929–34. 10.1172/JCI12749 11306593 PMC199563

[51] Carlson CB, Lawler J, Mosher DF. Structures of thrombospondins. Cell Mol Life Sci. 2008; 65:672–86. 10.1007/s00018-007-7484-1 18193164 PMC2578829

[52] Narouz-Ott L, Maurer P, Nitsche DP, Smyth N, Paulsson M. Thrombospondin-4 binds specifically to both collagenous and non-collagenous extracellular matrix proteins via its C-terminal domains. J Biol Chem. 2000; 275:37110–7. 10.1074/jbc.M007223200 10956668

[53] Adams JC. Functions of the conserved thrombospondin carboxy-terminal cassette in cell-extracellular matrix interactions and signaling. Int J Biochem Cell Biol. 2004; 36:1102–14. 10.1016/j.biocel.2004.01.022 15094125

[54] Stenina OI, Desai SY, Krukovets I, Kight K, Janigro D, Topol EJ, Plow EF. Thrombospondin-4 and its variants: expression and differential effects on endothelial cells. Circulation. 2003; 108:1514–9. 10.1161/01.CIR.0000089085.76320.4E 12952849

[55] Yang B, Liu Y, Zhao J, Hei K, Zhuang H, Li Q, Wei W, Chen R, Zhang N, Li Y. Ectopic overexpression of filamin C scaffolds MEK1/2 and ERK1/2 to promote the progression of human hepatocellular carcinoma. Cancer Lett. 2017; 388:167–76. 10.1016/j.canlet.2016.11.037 27919788

[56] Zhang Y, Li J, Lai XN, Jiao XQ, Xiong JP, Xiong LX. Focus on Cdc42 in Breast Cancer: New Insights, Target Therapy Development and Non-Coding RNAs. Cells. 2019; 8:146. 10.3390/cells8020146 30754684 PMC6406589

[57] Mayakonda A, Lin DC, Assenov Y, Plass C, Koeffler HP. Maftools: efficient and comprehensive analysis of somatic variants in cancer. Genome Res. 2018; 28:1747–56. 10.1101/gr.239244.118 30341162 PMC6211645

[58] Kurahara H, Shinchi H, Mataki Y, Maemura K, Noma H, Kubo F, Sakoda M, Ueno S, Natsugoe S, Takao S. Significance of M2-polarized tumor-associated macrophage in pancreatic cancer. J Surg Res. 2011; 167:e211–9. 10.1016/j.jss.2009.05.026 19765725

[59] Şenbabaoğlu Y, Michailidis G, Li JZ. Critical limitations of consensus clustering in class discovery. Sci Rep. 2014; 4:6207. 10.1038/srep06207 25158761 PMC4145288

[60] Hänzelmann S, Castelo R, Guinney J. GSVA: gene set variation analysis for microarray and RNA-seq data. BMC Bioinformatics. 2013; 14:7. 10.1186/1471-2105-14-7 23323831 PMC3618321

[61] Smyth GK, Michaud J, Scott HS. Use of within-array replicate spots for assessing differential expression in microarray experiments. Bioinformatics. 2005; 21:2067–75. 10.1093/bioinformatics/bti270 15657102

[62] Yoshihara K, Shahmoradgoli M, Martínez E, Vegesna R, Kim H, Torres-Garcia W, Treviño V, Shen H, Laird PW, Levine DA, Carter SL, Getz G, Stemke-Hale K, et al. Inferring tumour purity and stromal and immune cell admixture from expression data. Nat Commun. 2013; 4:2612. 10.1038/ncomms3612 24113773 PMC3826632

[63] Charoentong P, Finotello F, Angelova M, Mayer C, Efremova M, Rieder D, Hackl H, Trajanoski Z. Pan-cancer Immunogenomic Analyses Reveal Genotype-Immunophenotype Relationships and Predictors of Response to Checkpoint Blockade. Cell Rep. 2017; 18:248–62. 10.1016/j.celrep.2016.12.019 28052254

[64] Shi J, Jiang D, Yang S, Zhang X, Wang J, Liu Y, Sun Y, Lu Y, Yang K. LPAR1, Correlated With Immune Infiltrates, Is a Potential Prognostic Biomarker in Prostate Cancer. Front Oncol. 2020; 10:846. 10.3389/fonc.2020.00846 32656075 PMC7325998

[65] He Y, Jiang Z, Chen C, Wang X. Classification of triple-negative breast cancers based on Immunogenomic profiling. J Exp Clin Cancer Res. 2018; 37:327. 10.1186/s13046-018-1002-1 30594216 PMC6310928

[66] Auslander N, Zhang G, Lee JS, Frederick DT, Miao B, Moll T, Tian T, Wei Z, Madan S, Sullivan RJ, Boland G, Flaherty K, Herlyn M, Ruppin E. Robust prediction of response to immune checkpoint blockade therapy in metastatic melanoma. Nat Med. 2018; 24:1545–9. 10.1038/s41591-018-0157-9 30127394 PMC6693632

[67] Skidmore ZL, Wagner AH, Lesurf R, Campbell KM, Kunisaki J, Griffith OL, Griffith M. GenVisR: Genomic Visualizations in R. Bioinformatics. 2016; 32:3012–4. 10.1093/bioinformatics/btw325 27288499 PMC5039916

[68] Le DT, Uram JN, Wang H, Bartlett BR, Kemberling H, Eyring AD, Skora AD, Luber BS, Azad NS, Laheru D, Biedrzycki B, Donehower RC, Zaheer A, et al. PD-1 Blockade in Tumors with Mismatch-Repair Deficiency. N Engl J Med. 2015; 372:2509–20. 10.1056/NEJMoa1500596 26028255 PMC4481136

[69] Thorsson V, Gibbs DL, Brown SD, Wolf D, Bortone DS, Ou Yang TH, Porta-Pardo E, Gao GF, Plaisier CL, Eddy JA, Ziv E, Culhane AC, Paull EO, et al. The Immune Landscape of Cancer. Immunity. 2018; 48:812–30.e14. 10.1016/j.immuni.2018.03.023 Erratum in: Immunity. 2019; 51:411–2. 10.1016/j.immuni.2018.03.023 29628290 PMC5982584

[70] Rooney MS, Shukla SA, Wu CJ, Getz G, Hacohen N. Molecular and genetic properties of tumors associated with local immune cytolytic activity. Cell. 2015; 160:48–61. 10.1016/j.cell.2014.12.033 25594174 PMC4856474

[71] Zhang H, Meltzer P, Davis S. RCircos: an R package for Circos 2D track plots. BMC Bioinformatics. 2013; 14:244. 10.1186/1471-2105-14-244 23937229 PMC3765848

[72] Jiang P, Gu S, Pan D, Fu J, Sahu A, Hu X, Li Z, Traugh N, Bu X, Li B, Liu J, Freeman GJ, Brown MA, et al. Signatures of T cell dysfunction and exclusion predict cancer immunotherapy response. Nat Med. 2018; 24:1550–8. 10.1038/s41591-018-0136-1 30127393 PMC6487502

[73] Hoshida Y, Brunet JP, Tamayo P, Golub TR, Mesirov JP. Subclass mapping: identifying common subtypes in independent disease data sets. PLoS One. 2007; 2:e1195. 10.1371/journal.pone.0001195 18030330 PMC2065909

[74] Ji Q, Huang K, Jiang Y, Lei K, Tu Z, Luo H, Zhu X. Comprehensive analysis of the prognostic and role in immune cell infiltration of MSR1 expression in lower-grade gliomas. Cancer Med. 2022; 11:2020–35. 10.1002/cam4.4603 35142109 PMC9089222

