# Supplementary material for: An integrated bioinformatic investigation of focal adhesion-related genes in glioma followed by preliminary validation of COL1A2 in tumorigenesis
Source: Aging (Albany NY). 2023 Jun 22;15(13):6225–54. doi: 10.18632/aging.204834 (PMC10373961; doi:10.18632/aging.204834)
Supplement: Supplementary Figures [file aging-15-204834-s001.pdf]

SUPPLEMENTARY FIGURES

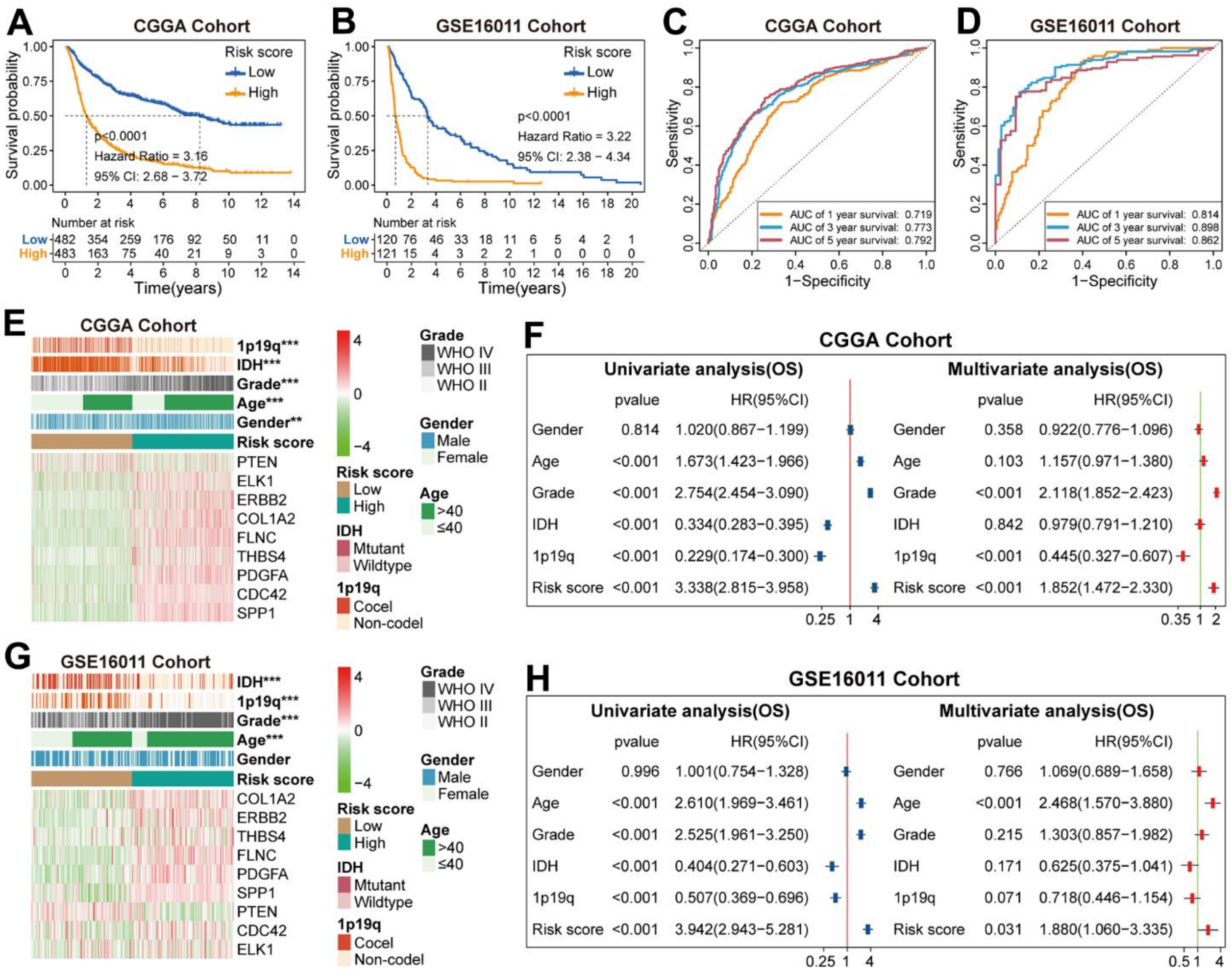

Supplementary Figure 1. Validation of prognostic value of the scoring system in CGGA and GSE16011 cohorts. (**A**, **B**) Kaplan-Meier curves of the risk score for the OS of glioma patients between the high and low-risk groups. (**C**, **D**) ROC curves verified the accuracy of the risk score in predicting the OS for glioma patients. (**E**) The distributions of five clinicopathological characteristics and nine FARGs between high and low-risk groups in the CGGA cohort. (**F**) Univariate and multivariate Cox regression analysis of risk score and five clinicopathological characteristics in the CGGA cohort. (**G**) The distributions of five clinicopathological characteristics and nine FARGs between high and low-risk groups in the GSE16011 cohort. (**H**) Univariate and multivariate Cox regression analysis of risk score and five clinicopathological characteristics in the GSE16011 cohort. *P<0.05, **P<0.01, ***P<0.001.

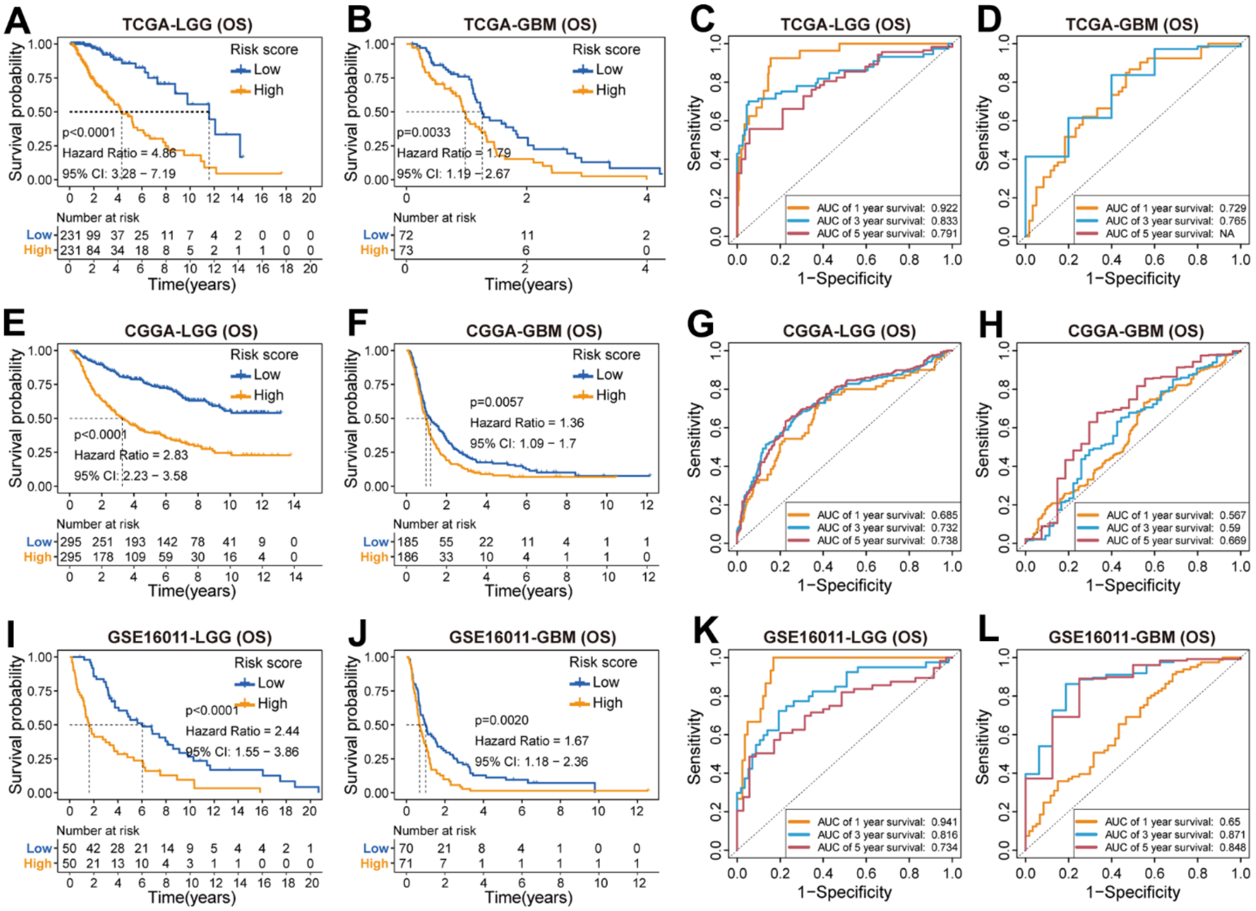

Supplementary Figure 2. Prognostic analysis of the scoring system in TCGA, CGGA, and GSE16011 cohorts. (**A**, **B**) K-M curves of the risk score in predicting the OS of LGG and GBM patients between the high and low-risk groups in the TCGA cohort. (**C**, **D**) ROC curves verified the accuracy of the risk score in predicting the OS for LGG and GBM patients in the TCGA cohort. (**E**, **F**) K-M curves of the risk score in predicting the OS of LGG and GBM patients between the high and low-risk groups in the CGGA cohort. (**G**, **H**) ROC curves verified the accuracy of the risk score in predicting the OS for LGG and GBM patients in the CGGA cohort. (**I**, **J**) K-M curves of the risk score in predicting the OS of LGG and GBM patients between the high and low-risk groups in the GSE16011 cohort. (**K**, **L**) ROC curves verified the accuracy of the risk score in predicting the OS for LGG and GBM patients in the GSE16011 cohort.

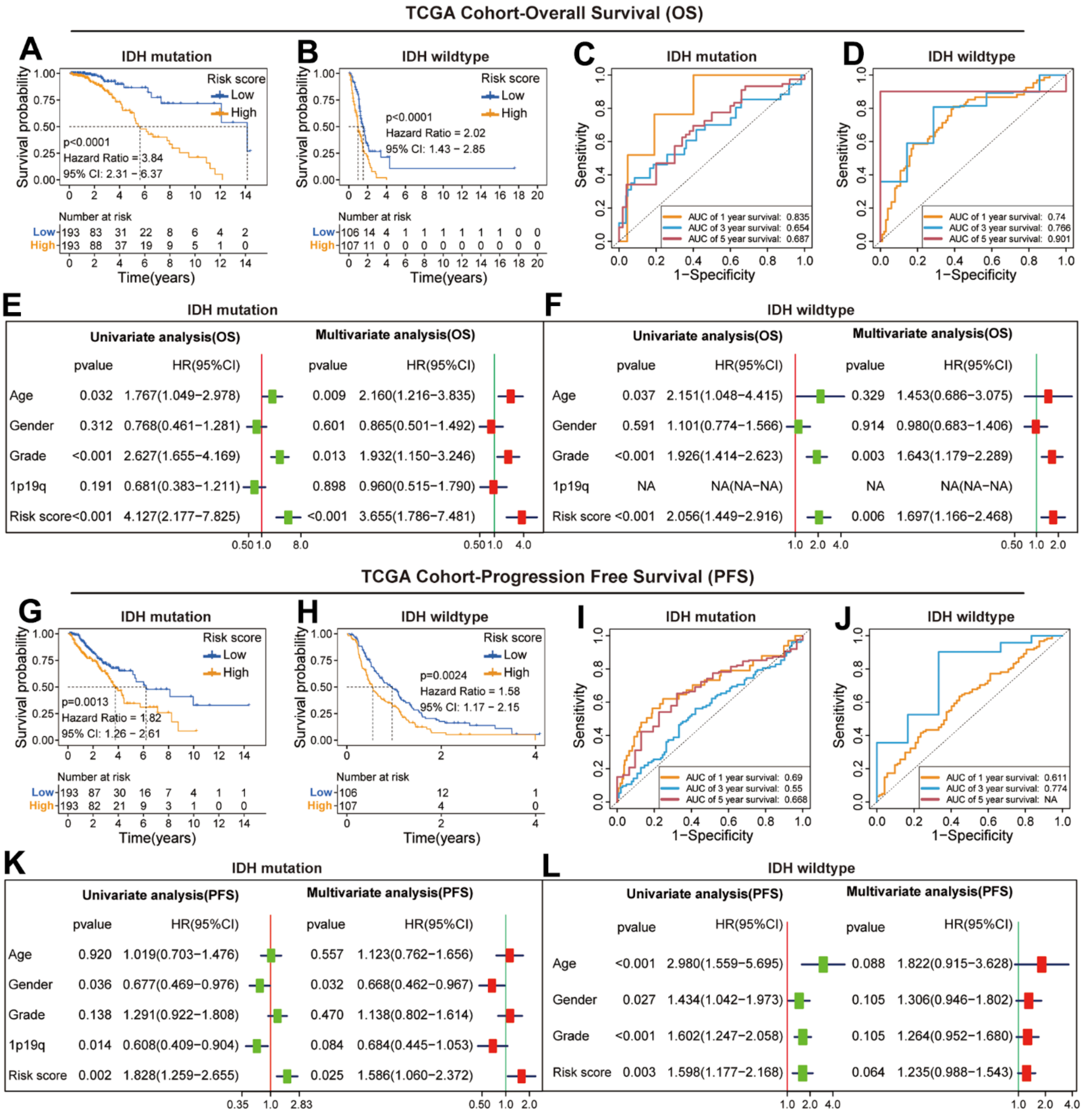

Supplementary Figure 3. Prognostic analysis of risk score in glioma patients with IDH mutation or wildtype in the TCGA cohort. (**A**, **B**) K-M survival curves of the risk score in predicting the OS for glioma patients with IDH mutation or wildtype between the high and low-risk groups. (**C**, **D**) ROC curves verified the accuracy of the risk score in predicting the OS for glioma patients with IDH mutation or wildtype. (**E**, **F**) Univariate and multivariate-Cox regression analysis of risk score, 1p19q status, grade, gender, and age. (**G**, **H**) K-M survival curves of the risk score in predicting the PFS for glioma patients with IDH mutation or wildtype between the high and low-risk groups. (**I**, **J**) ROC curves verified the accuracy of the risk score in predicting the PFS for glioma patients with IDH mutation or wildtype. (**K**, **L**) Univariate and multivariate-Cox regression analysis of risk score, 1p19q status, grade, gender, and age.

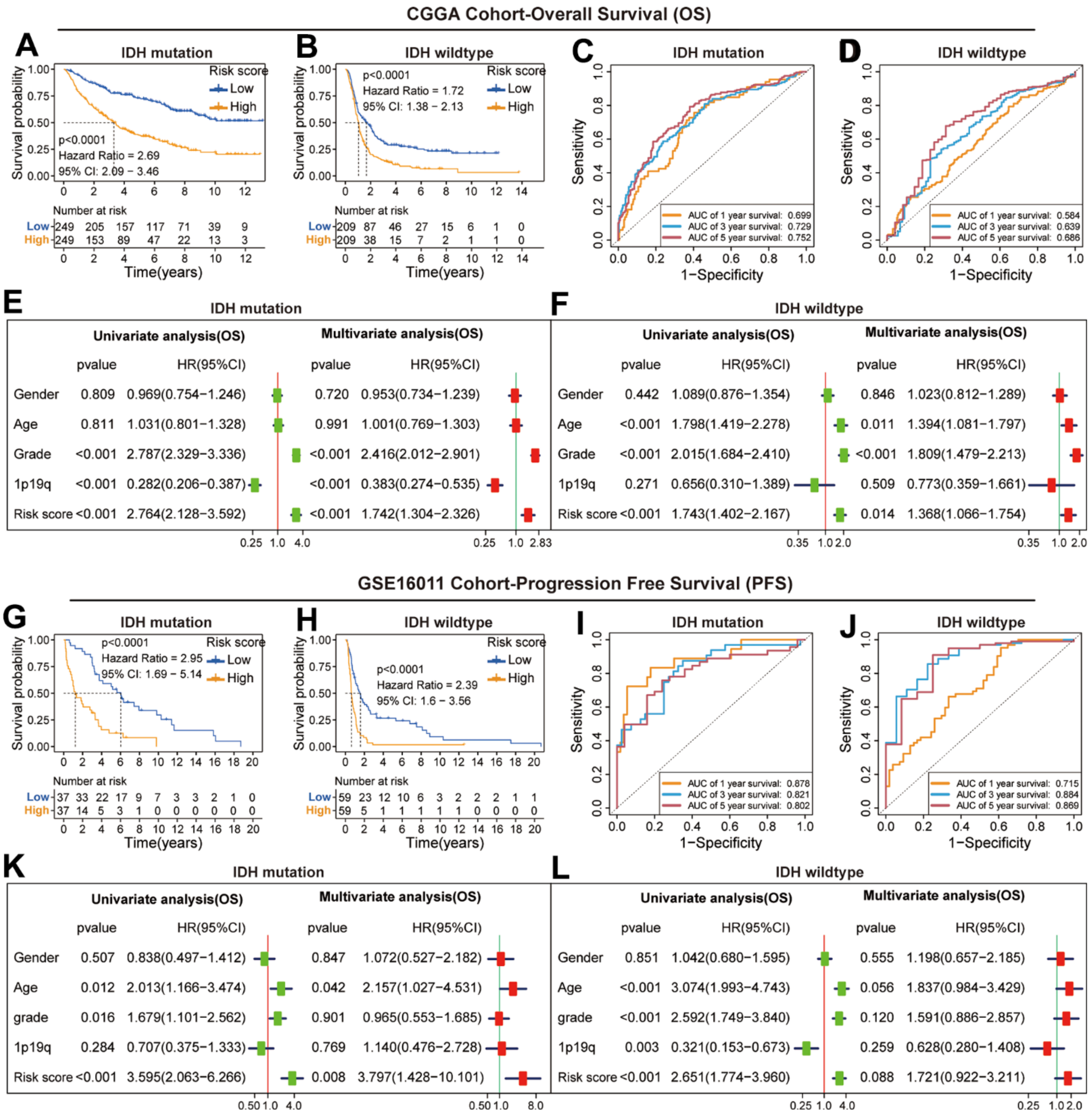

Supplementary Figure 4. Prognostic analysis of risk score in glioma patients with IDH mutation or wildtype in CGGA and GSE16011 cohorts. (**A**, **B**) K-M survival curves of the risk score in predicting the OS for glioma patients with IDH mutation or wildtype between the high- and low-risk groups in the CGGA cohort. (**C**, **D**) ROC curves verified the accuracy of the risk score in predicting the OS for glioma patients with IDH mutation or wildtype in the CGGA cohort. (**E**, **F**) Univariate and multivariate-Cox regression analysis of risk score, 1p19q status, grade, gender, and age. (**G**, **H**) K-M survival curves of the risk score in predicting the OS for glioma patients with IDH mutation or wildtype between the high- and low-risk groups in the GSE16011 cohort. (**I**, **J**) ROC curves verified the accuracy of the risk score in predicting the OS for glioma patients with IDH mutation or wildtype in the GSE16011 cohort. (**K**, **L**) Univariate and multivariate-Cox regression analysis of risk score, 1p19q status, grade, gender, and age in the GSE16011 cohort.

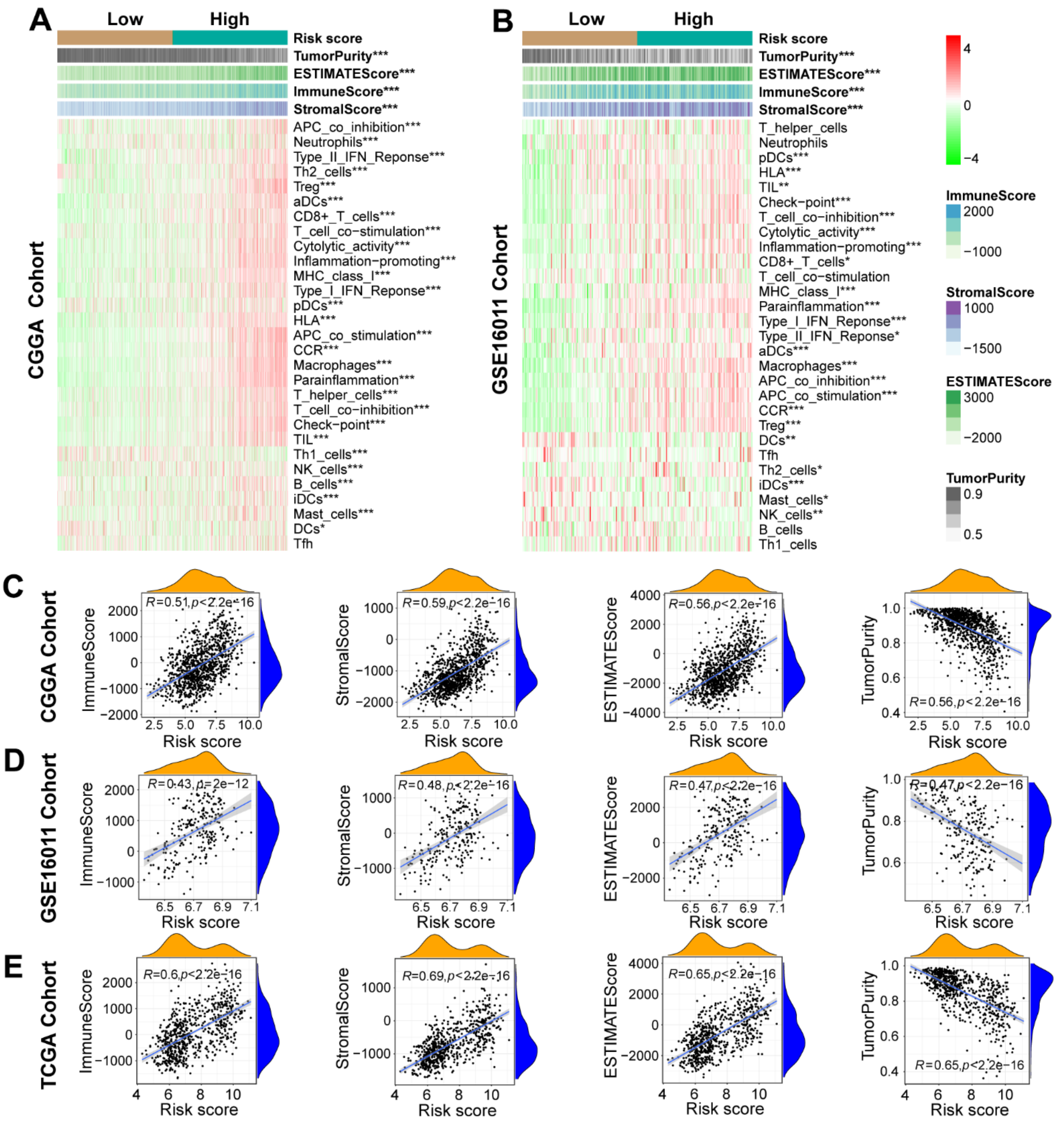

Supplementary Figure 5. Relationship between risk score and immune signatures and TME-related scores. (**A**, **B**) Analysis of the difference in enrichment score of immune signatures and TME-related scores between high and low-risk score groups in the CGGA (**A**) and GSE16011 (**B**) cohorts. (**C**–**E**) Scatter plots of the correlations between four types of TME-related scores and risk score in the TCGA (**E**), CGGA (**C**), and GSE16011 (**D**) cohorts. The blue lines represent the correlation and the black dots represent the glioma samples. *P<0.05, **P<0.01, ***P<0.001.

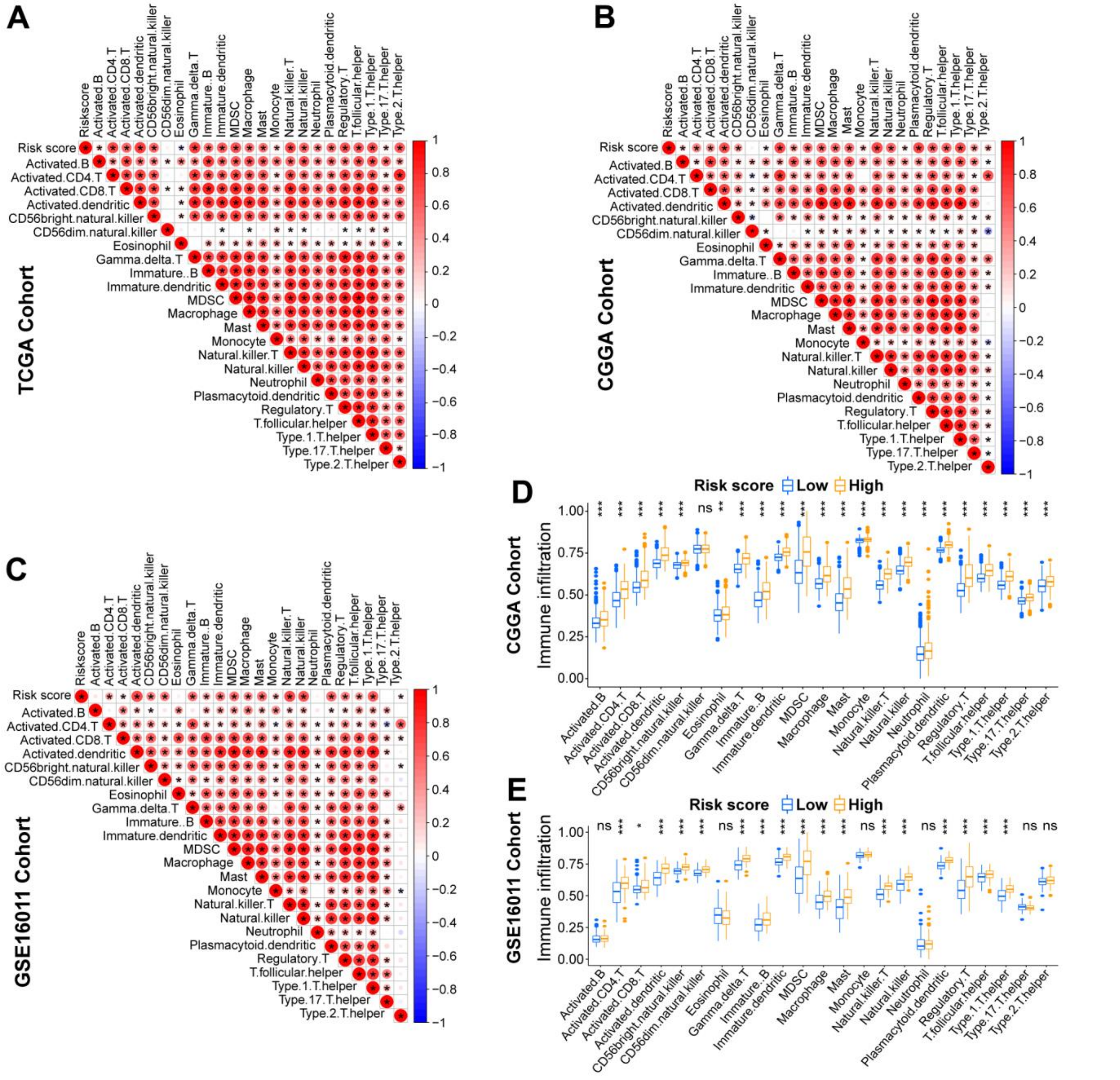

Supplementary Figure 6. The association between risk score and immune cells in the TCGA, CGGA, and GSE16011 cohorts. (**A**–**C**) Correlation between risk score and immune cells. A negative correlation is marked with blue and a positive correlation is marked with red. (**D**, **E**) Differences in the infiltration level of immune cells between high and low-risk groups in the CGGA and GSE16011 cohorts. *P<0.05, **P<0.01, ***P<0.001.

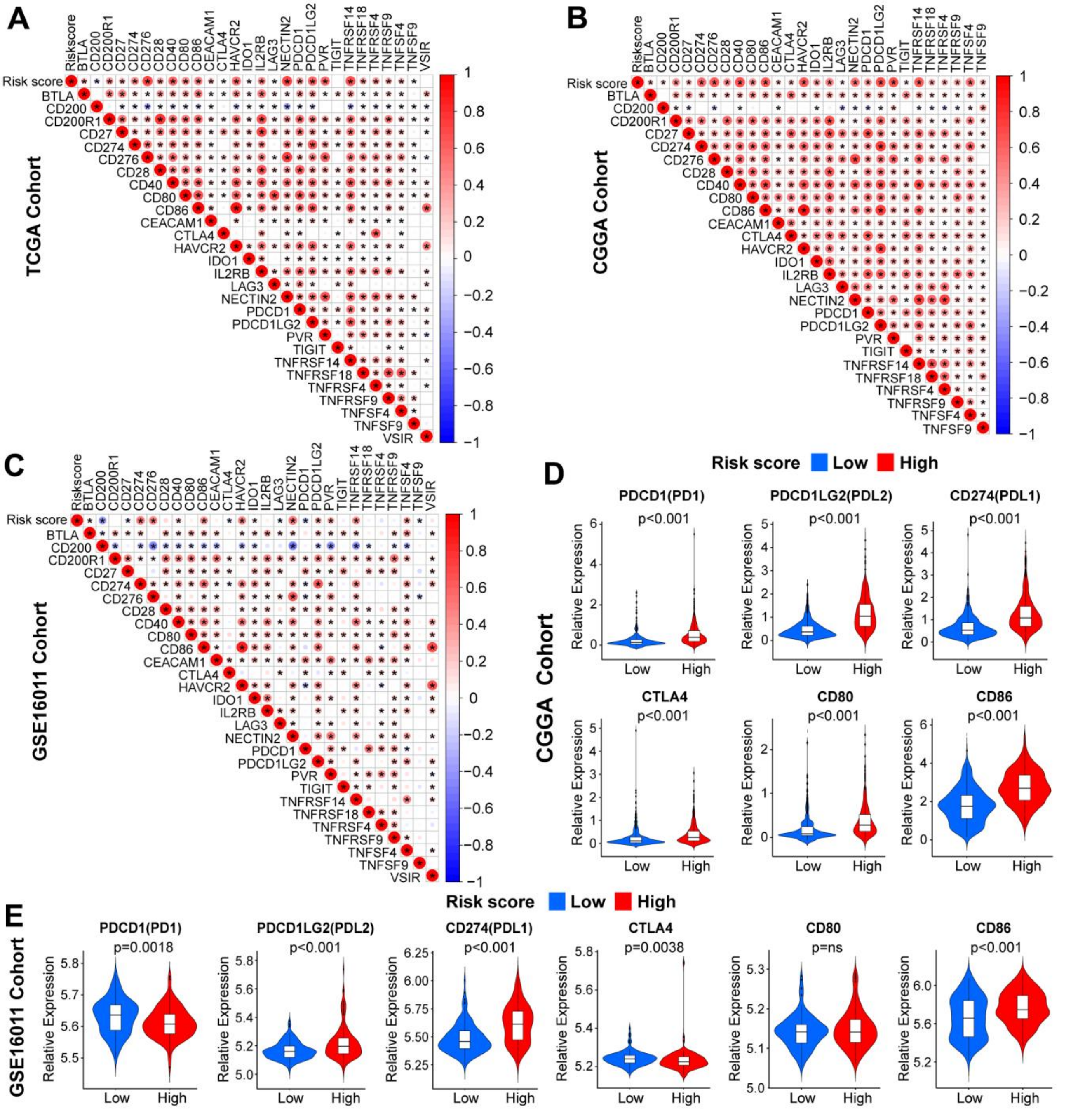

Supplementary Figure 7. The association between risk score and ICPs in the TCGA, CGGA, and GSE16011 cohorts. (**A**–**C**) Correlation between risk score and ICPs. A negative correlation is marked with blue and a positive correlation is marked with red. *P<0.05. (**D**, **E**) Differences in the relative expression level of six well-known ICPs between high and low-risk groups.

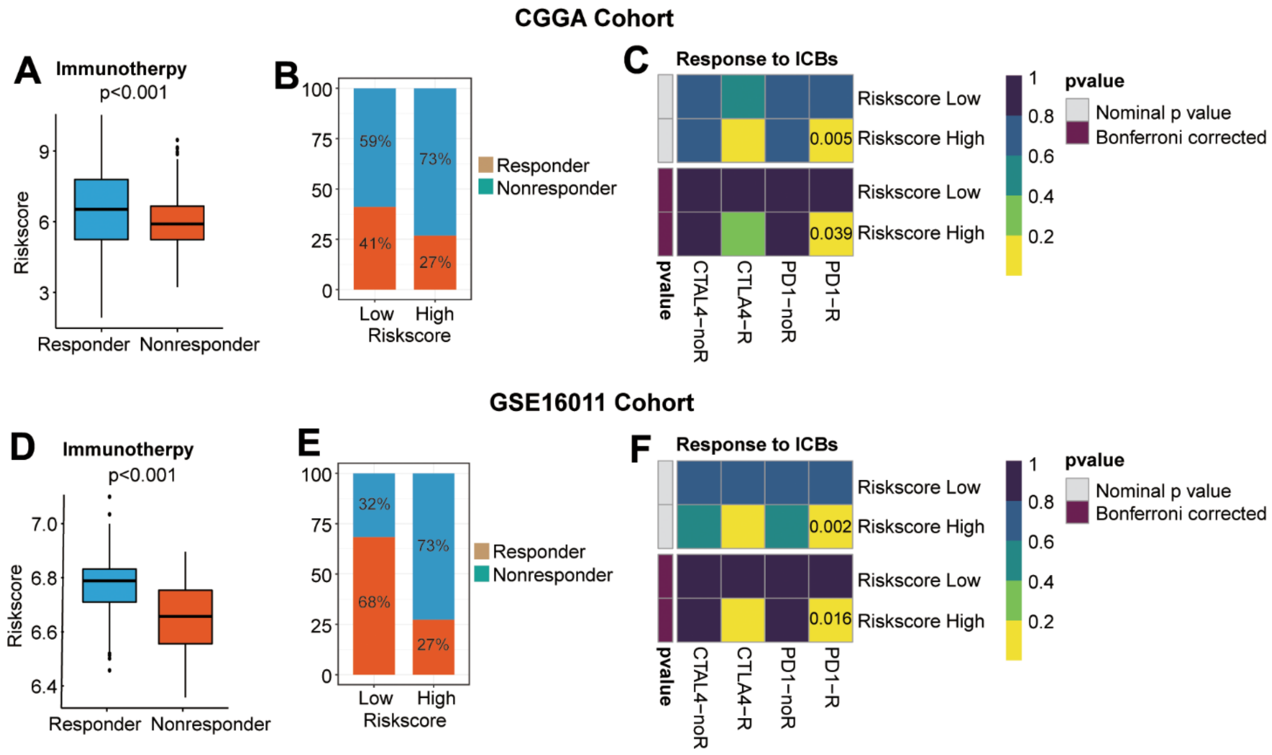

Supplementary Figure 8. Differential response to immunotherapy between high and low-risk groups in the CGGA and GSE16011 cohorts. (**A**) The difference in risk score between two immunotherapy response groups in the CGGA cohort. (**B**) The proportion of responders and non-responders to immunotherapy between high and low-risk groups in the CGGA cohort. (**C**) Subgroup map of predicted response to ICB therapy in the two risk groups in the CGGA cohort. (**D**) The difference in risk score between two immunotherapy response groups in the GSE16011 cohort. (**E**) The proportion of responders and non-responders to immunotherapy between high and low-risk groups in the GSE16011 cohort. (**F**) Subgroup map of predicted response to ICB therapy in the two risk groups in the GSE16011 cohort. In (**A**, **D**), the upper and lower lines of the boxes indicate the interquartile range of values, and the lines in the boxes represent the median value, and the black dots show outliers.
