# Supplementary material for: An integrated bioinformatic investigation of focal adhesion-related genes in glioma followed by preliminary validation of COL1A2 in tumorigenesis
Source: Aging (Albany NY). 2023 Jun 22;15(13):6225–54. doi: 10.18632/aging.204834 (PMC10373961; doi:10.18632/aging.204834)
Supplement: Supplementary Table 1 [file aging-15-204834-s002.pdf]

SUPPLEMENTARY TABLE

Supplementary Table 1. Demographics and clinical information of glioma patients in TCGA, CGGA and GSE16011 cohorts.

Variables	TCGA cohort Total(n=607)	CGGA cohort Total(n=965)	GSE16011 cohort Total(n=241)
OS Status			
Alive	408	358	23
Dead	199	607	218
Gender			
Female	259	398	81
Male	348	567	160
Age			
>40	372	561	174
<=40	235	403	67
NA	0	1	0
Grade			
II	222	270	20
III	240	322	80
IV	145	373	141
IDH			
Mutant	386	498	74
Wildtype	213	418	118
NA	8	49	49
1p19q			
Codel	153	199	43
Non-codel	450	695	97
NA	4	71	101
